# Supplementary material for: Partnerships at the Interface of Education and Mental Health Services: The Utilisation and Acceptability of the Provision of Specialist Liaison and Teacher Skills Training
Source: Int J Environ Res Public Health. 2023 Feb 24;20(5):4066. doi: 10.3390/ijerph20054066 (PMC10001585; doi:10.3390/ijerph20054066)
Supplement: Supplementary file 1 [file ijerph-20-04066-s001.zip › ijerph-2136677-supplementary.pptx]

## Slide 1
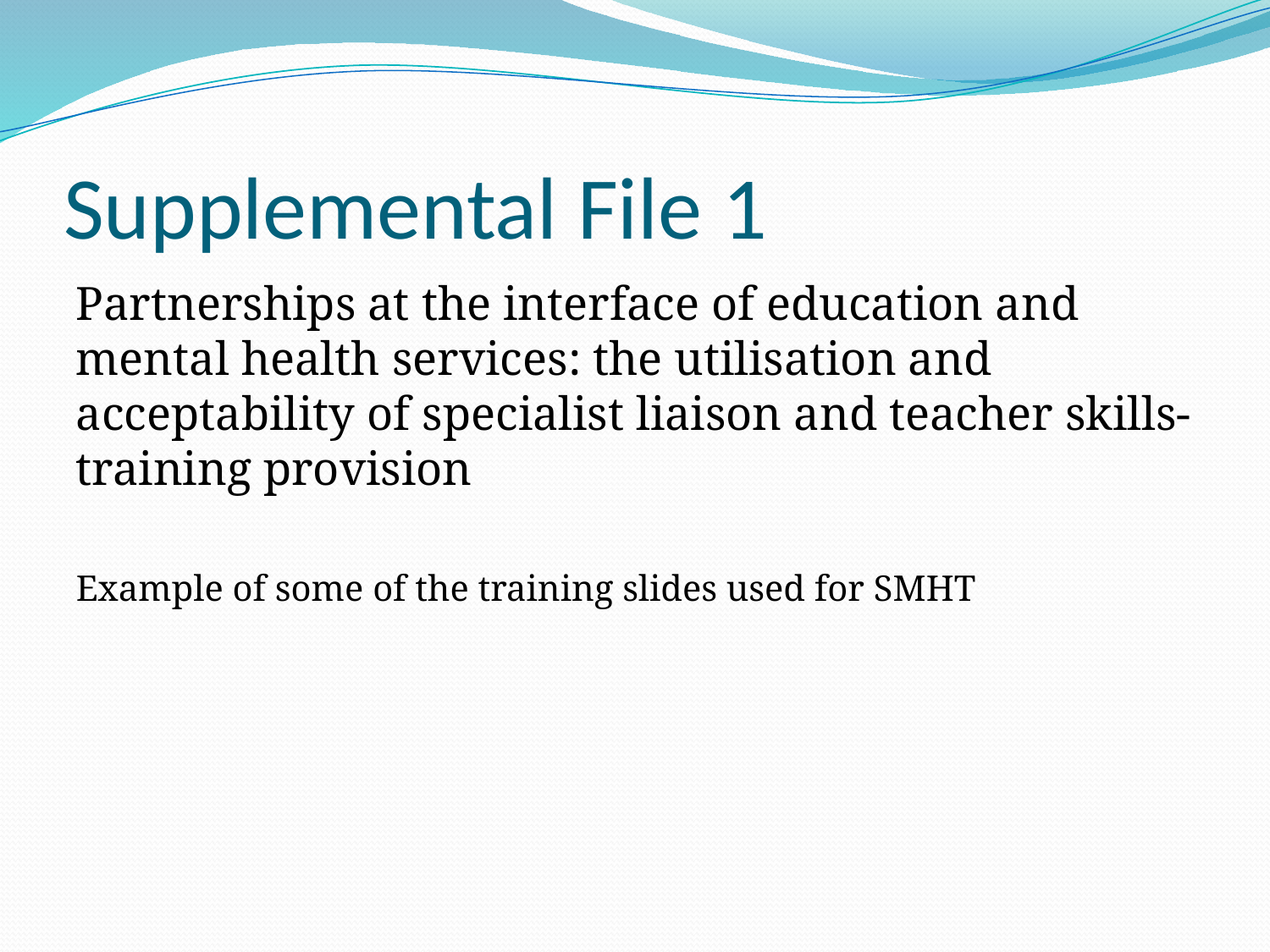

# Supplemental File 1
Partnerships at the interface of education and mental health services: the utilisation and acceptability of specialist liaison and teacher skills-training provision
Example of some of the training slides used for SMHT

## Slide 2
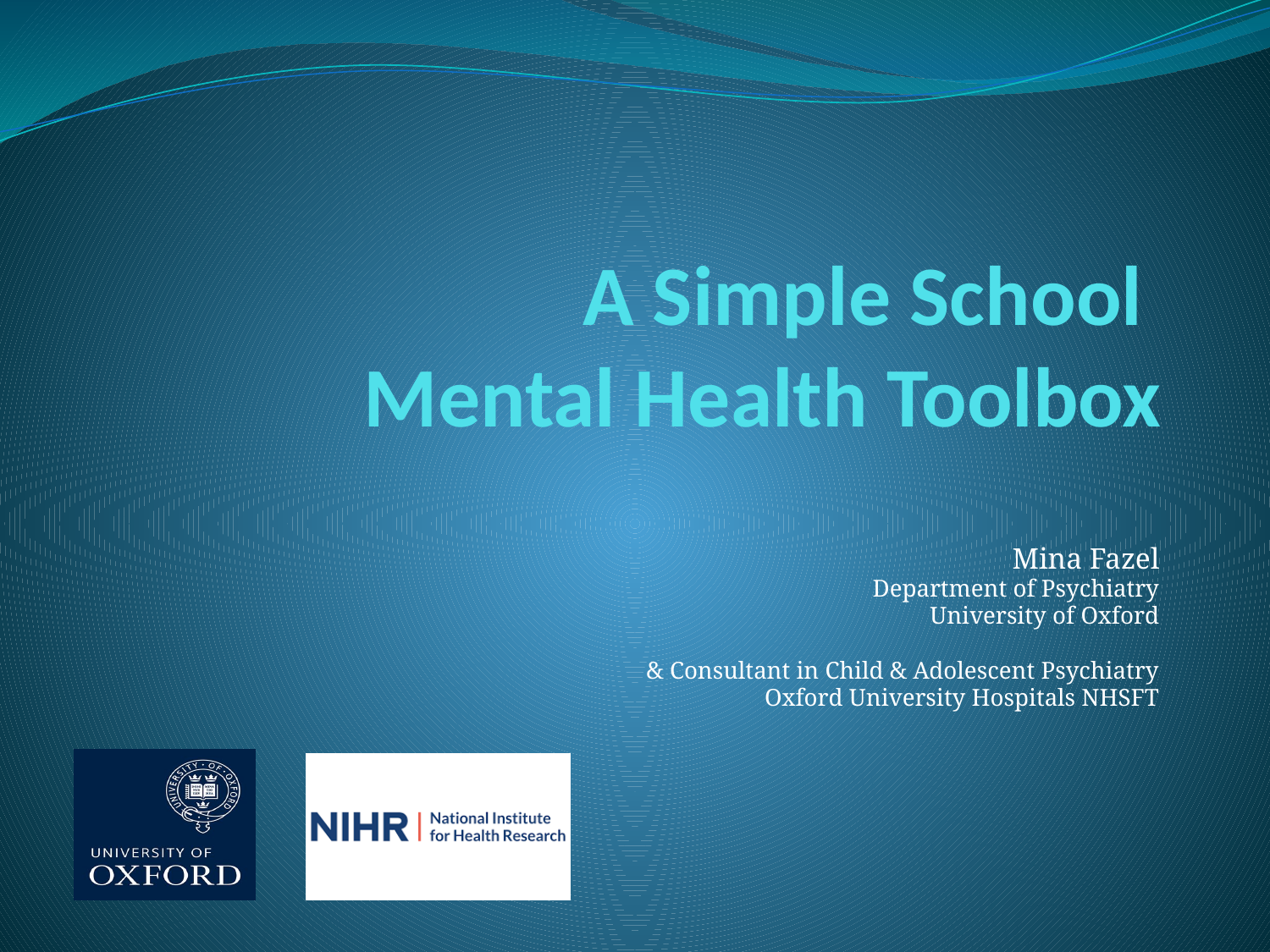

# A Simple School Mental Health Toolbox
Mina Fazel
Department of Psychiatry
University of Oxford
& Consultant in Child & Adolescent Psychiatry
Oxford University Hospitals NHSFT

## Slide 3
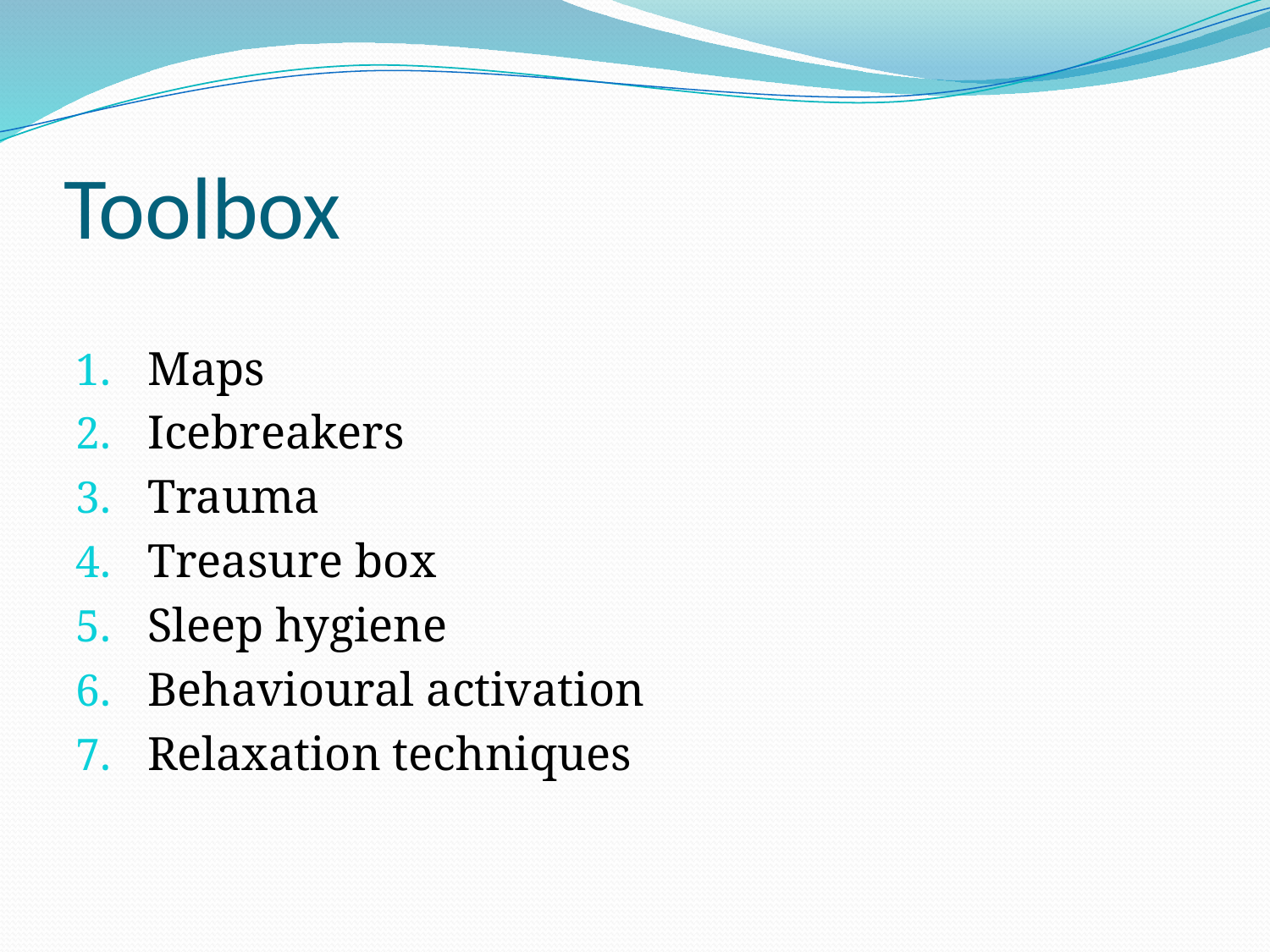

# Toolbox
Maps
Icebreakers
Trauma
Treasure box
Sleep hygiene
Behavioural activation
Relaxation techniques

## Slide 4
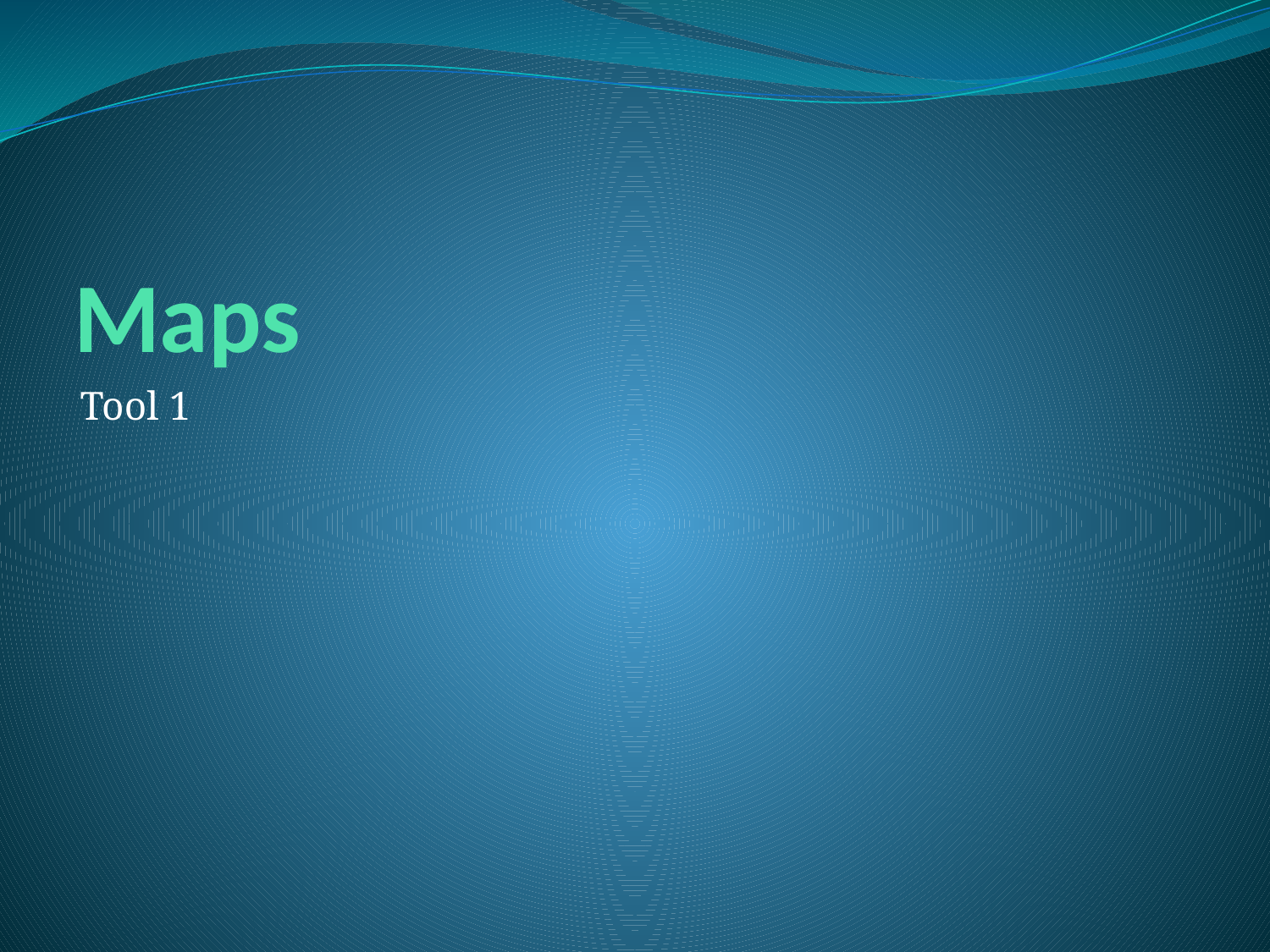

# Maps
Tool 1

## Slide 5
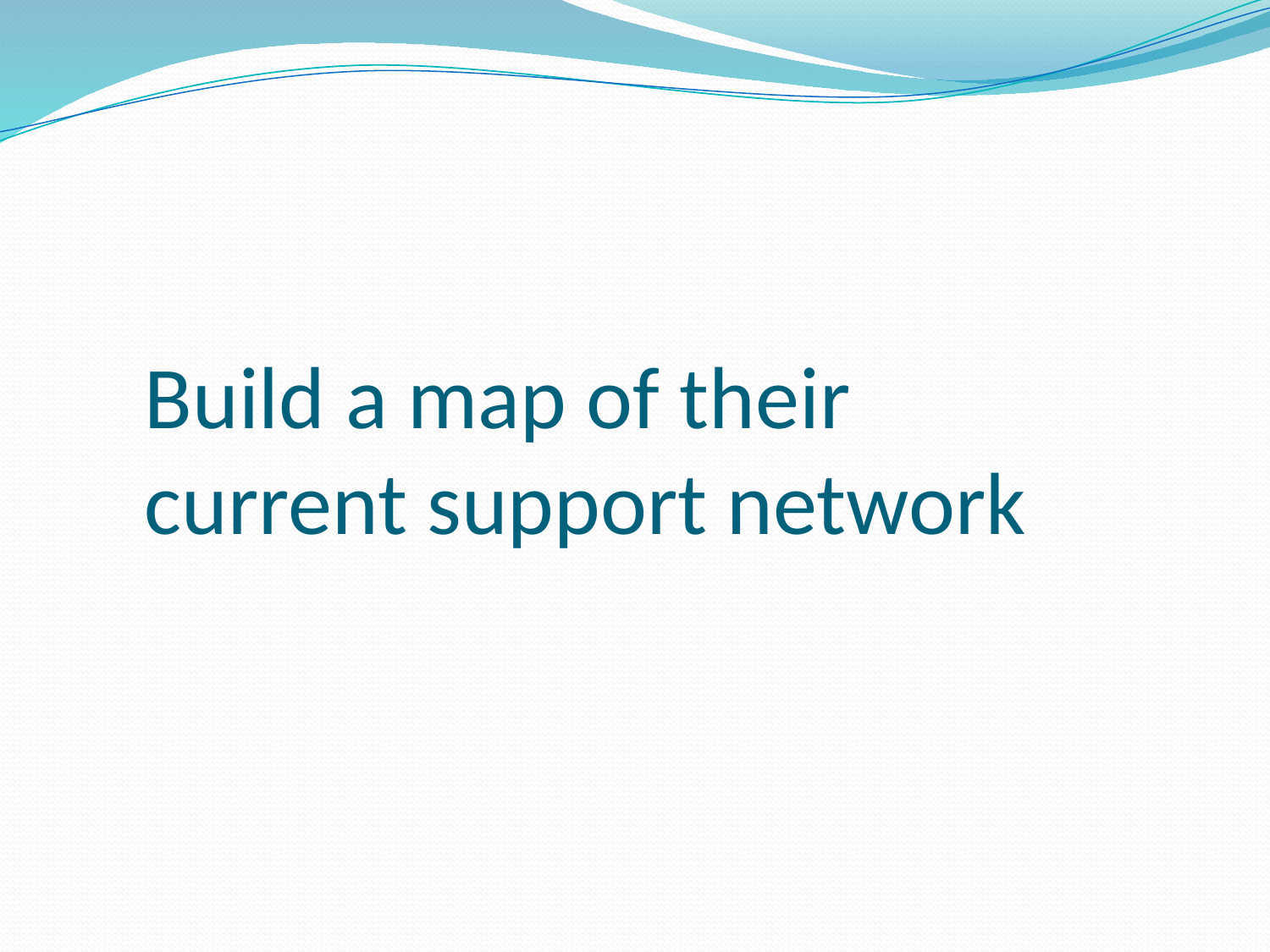

# Build a map of their current support network

## Slide 6
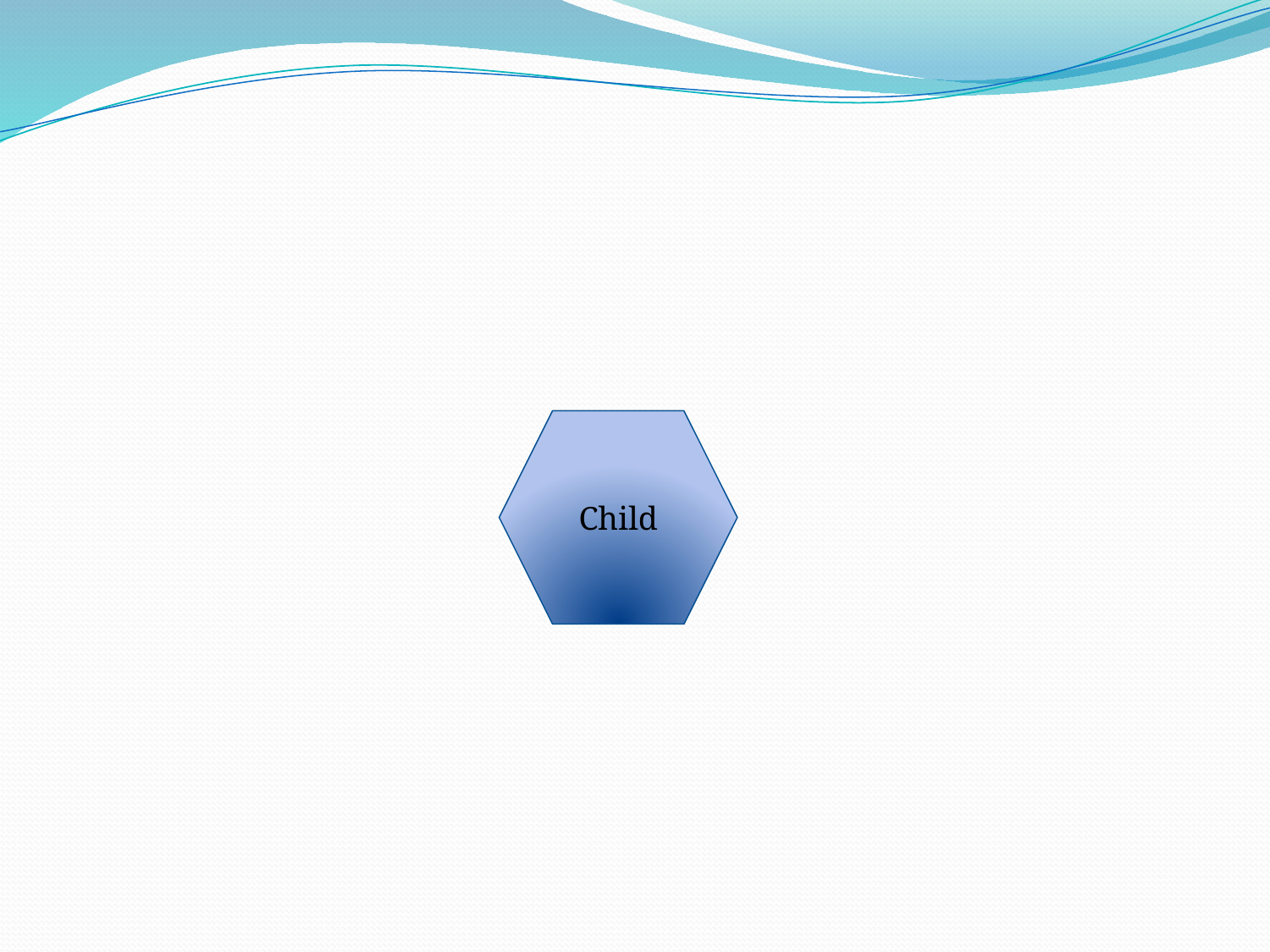

Child

## Slide 7
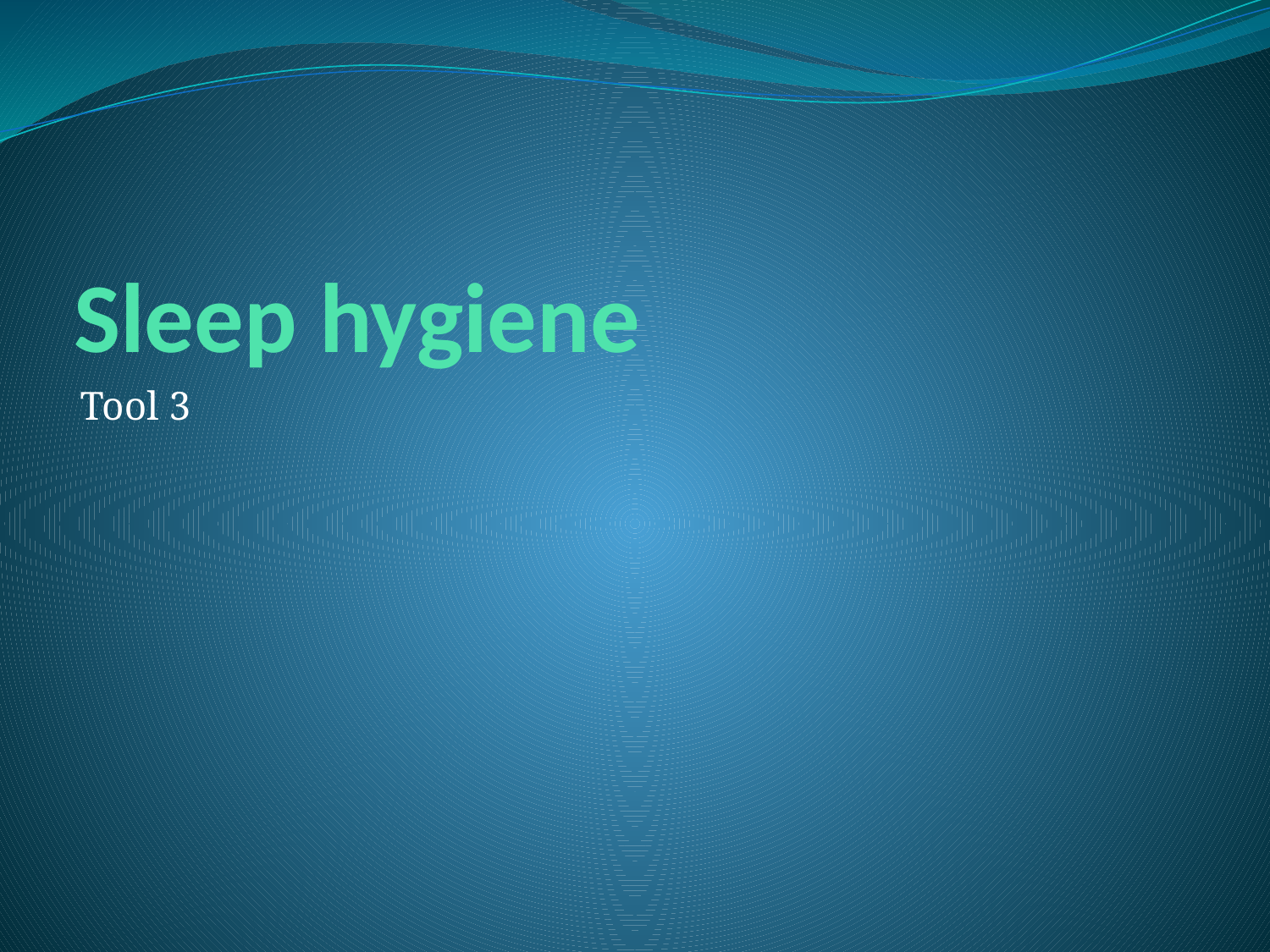

# Sleep hygiene
Tool 3

## Slide 8
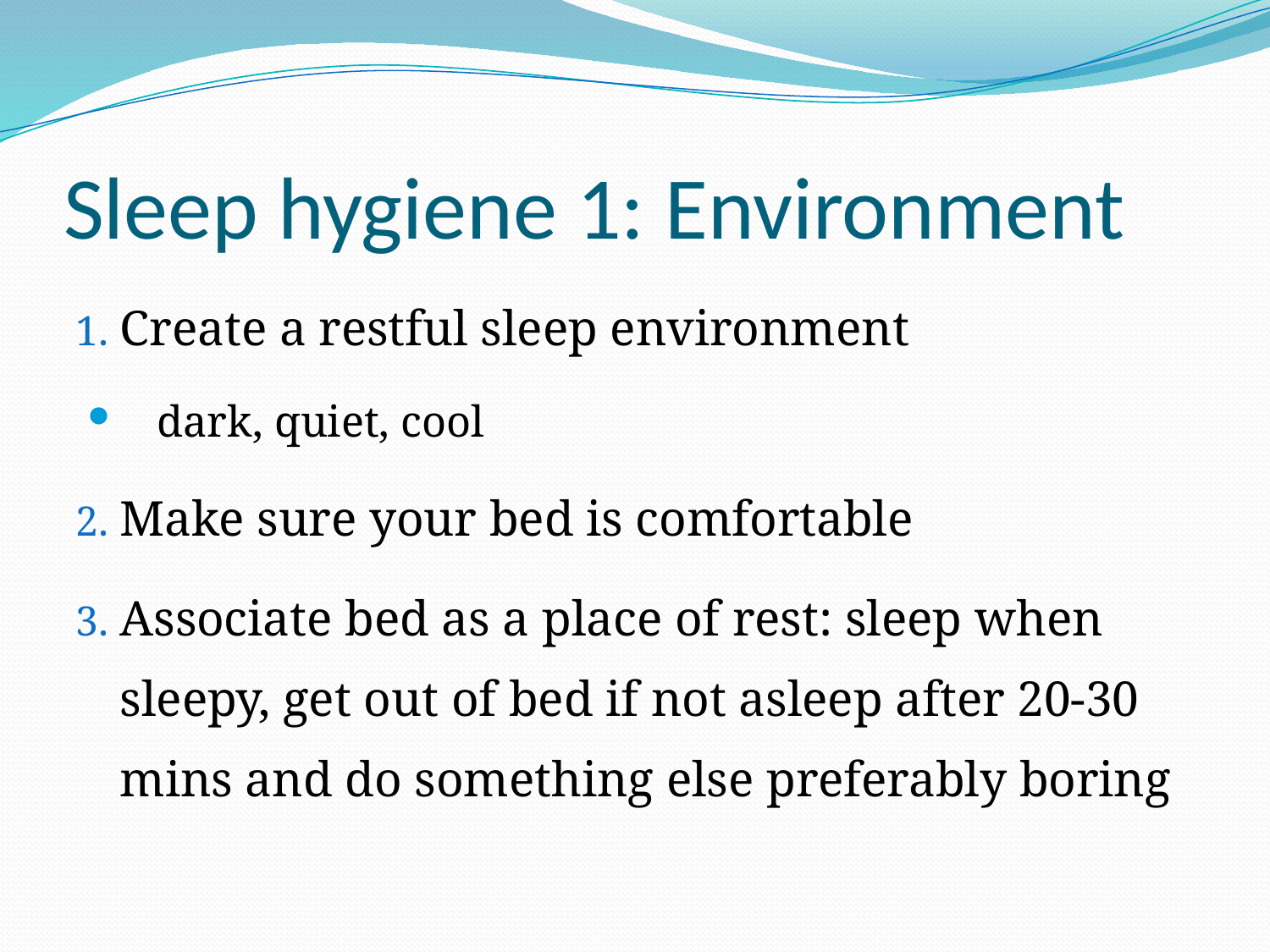

# Sleep hygiene 1: Environment
Create a restful sleep environment
dark, quiet, cool
Make sure your bed is comfortable
Associate bed as a place of rest: sleep when sleepy, get out of bed if not asleep after 20-30 mins and do something else preferably boring

## Slide 9
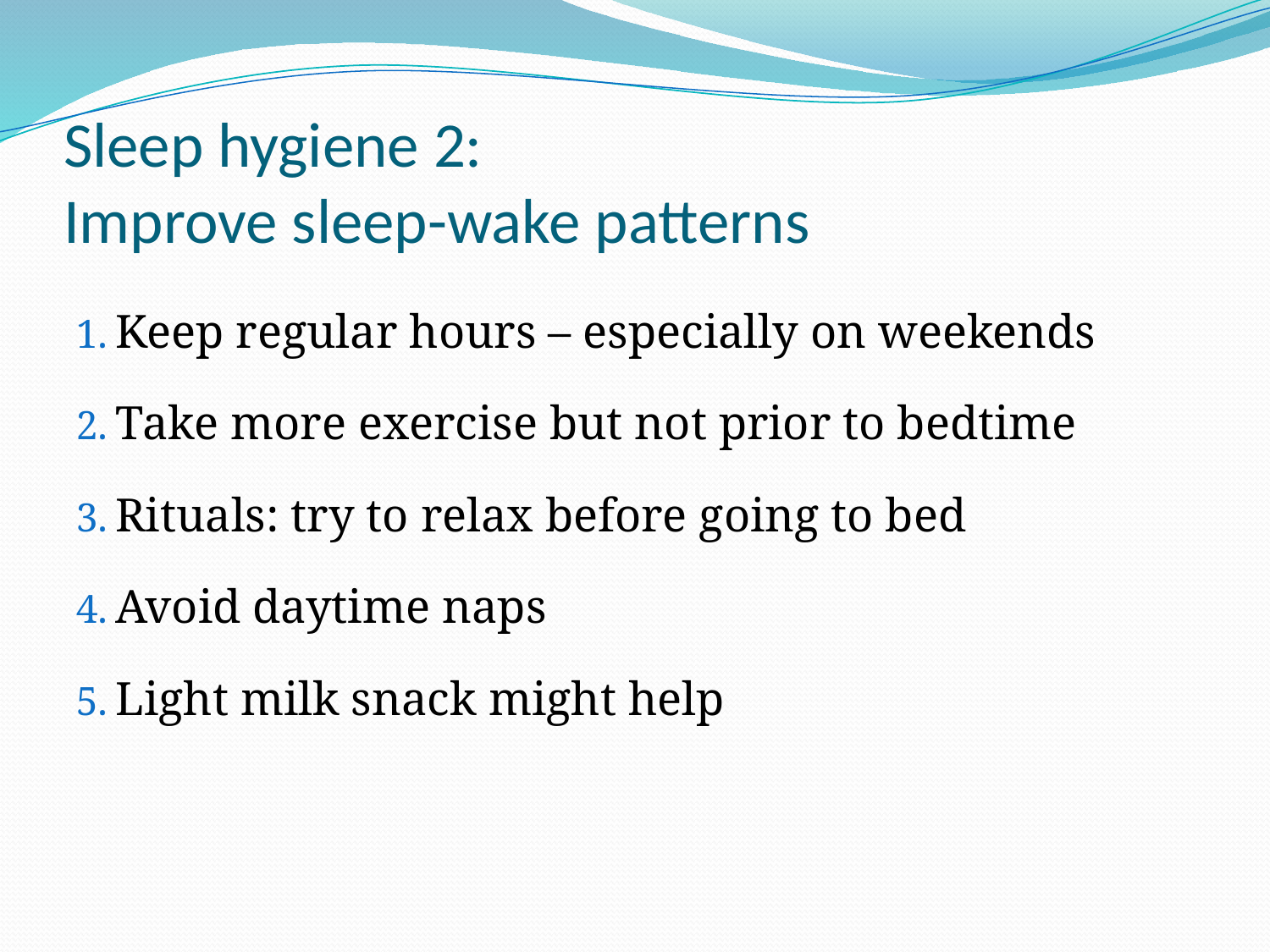

# Sleep hygiene 2: Improve sleep-wake patterns
Keep regular hours – especially on weekends
Take more exercise but not prior to bedtime
Rituals: try to relax before going to bed
Avoid daytime naps
Light milk snack might help

## Slide 10
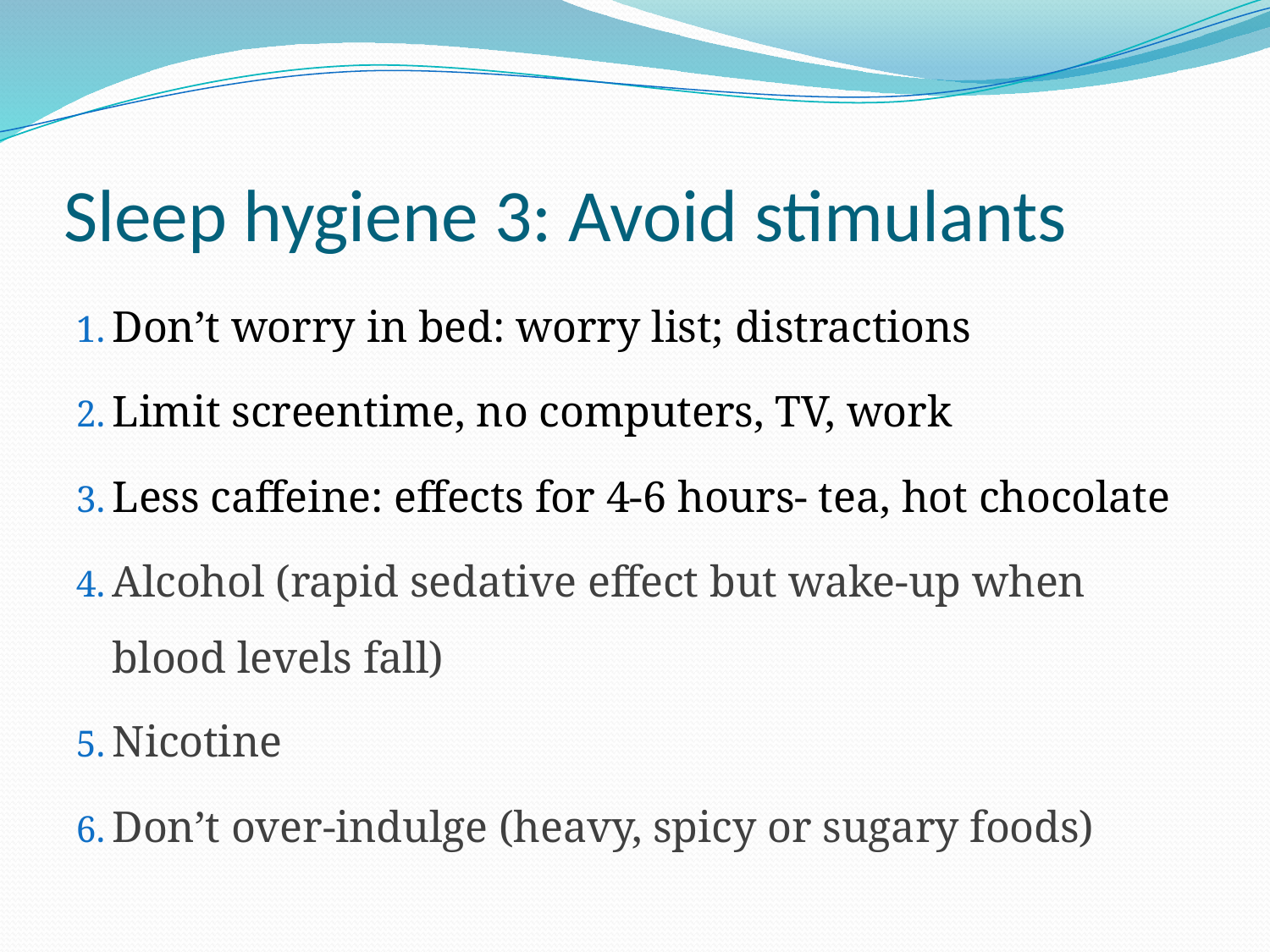

# Sleep hygiene 3: Avoid stimulants
Don’t worry in bed: worry list; distractions
Limit screentime, no computers, TV, work
Less caffeine: effects for 4-6 hours- tea, hot chocolate
Alcohol (rapid sedative effect but wake-up when blood levels fall)
Nicotine
Don’t over-indulge (heavy, spicy or sugary foods)

## Slide 11
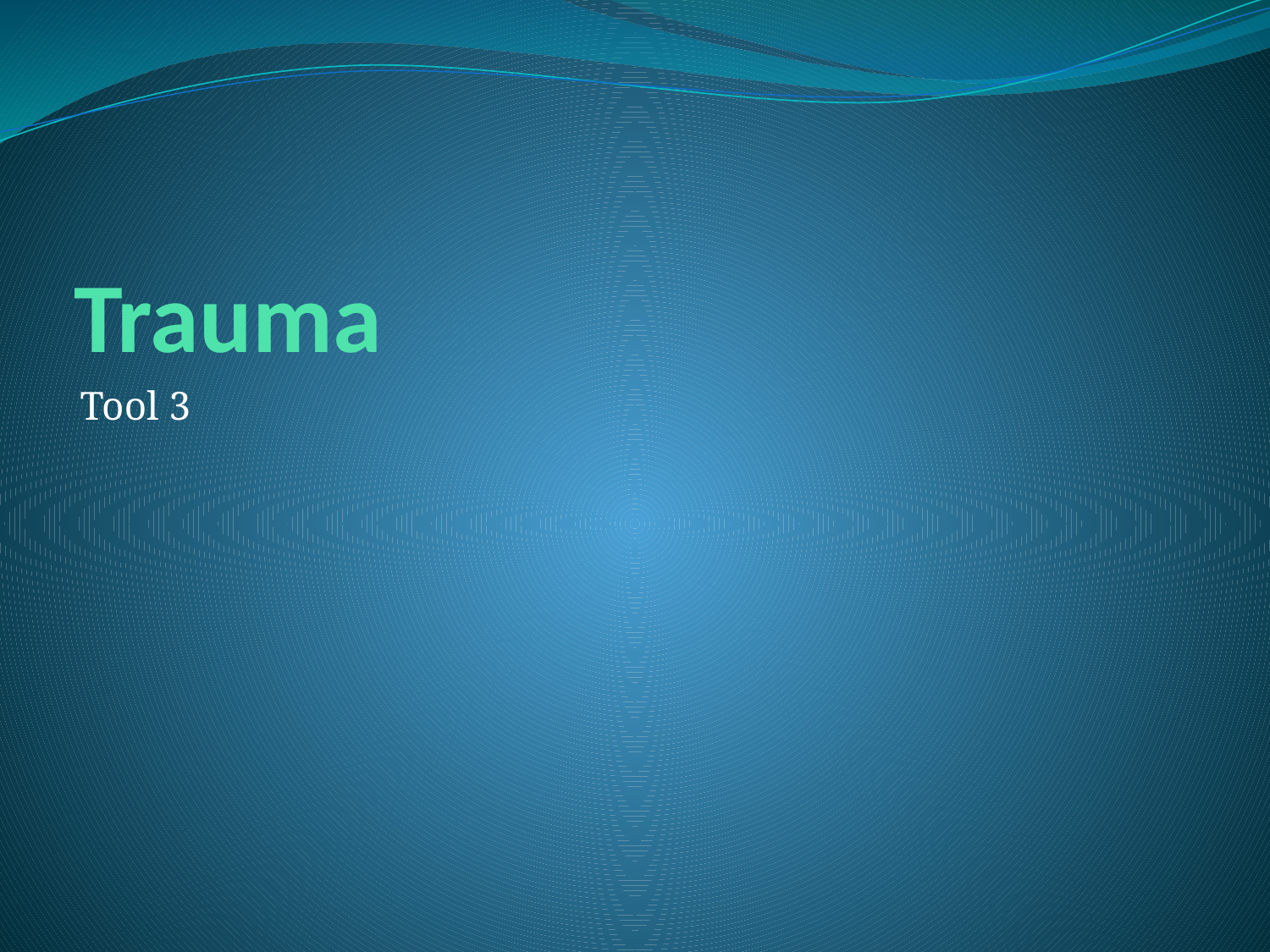

# Trauma
Tool 3

## Slide 12
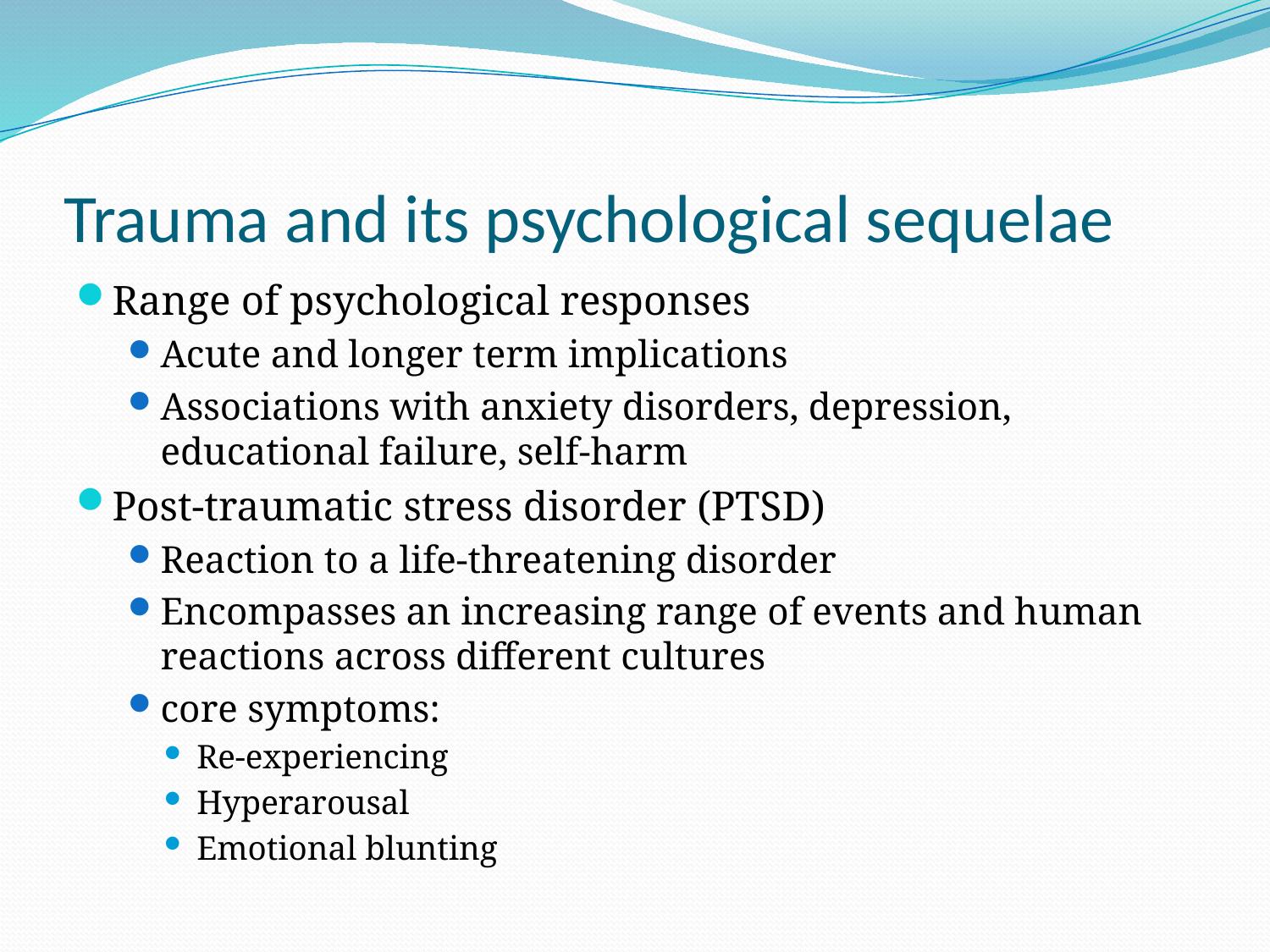

# Trauma and its psychological sequelae
Range of psychological responses
Acute and longer term implications
Associations with anxiety disorders, depression, educational failure, self-harm
Post-traumatic stress disorder (PTSD)
Reaction to a life-threatening disorder
Encompasses an increasing range of events and human reactions across different cultures
core symptoms:
Re-experiencing
Hyperarousal
Emotional blunting

## Slide 13
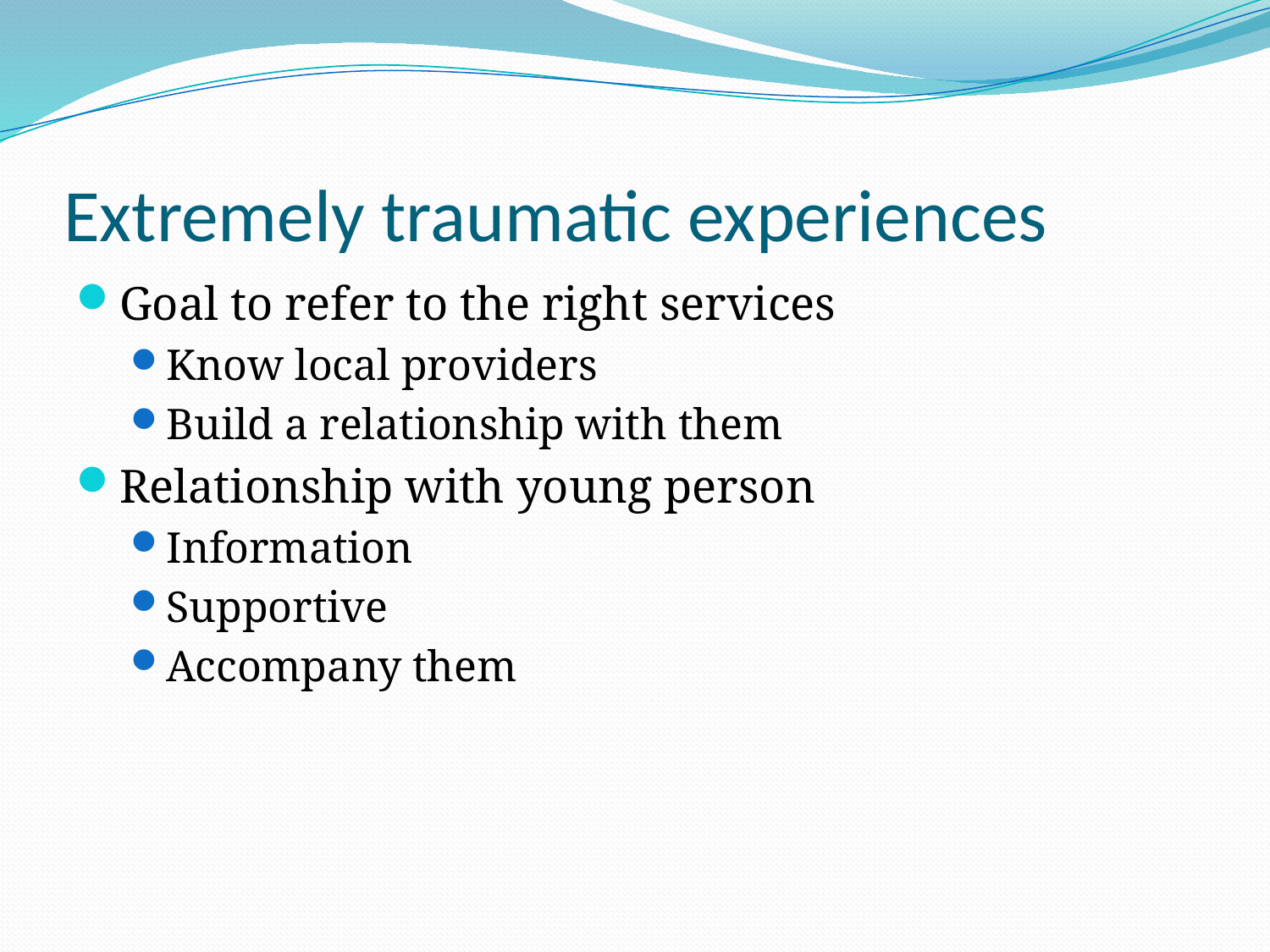

# Extremely traumatic experiences
Goal to refer to the right services
Know local providers
Build a relationship with them
Relationship with young person
Information
Supportive
Accompany them

## Slide 14
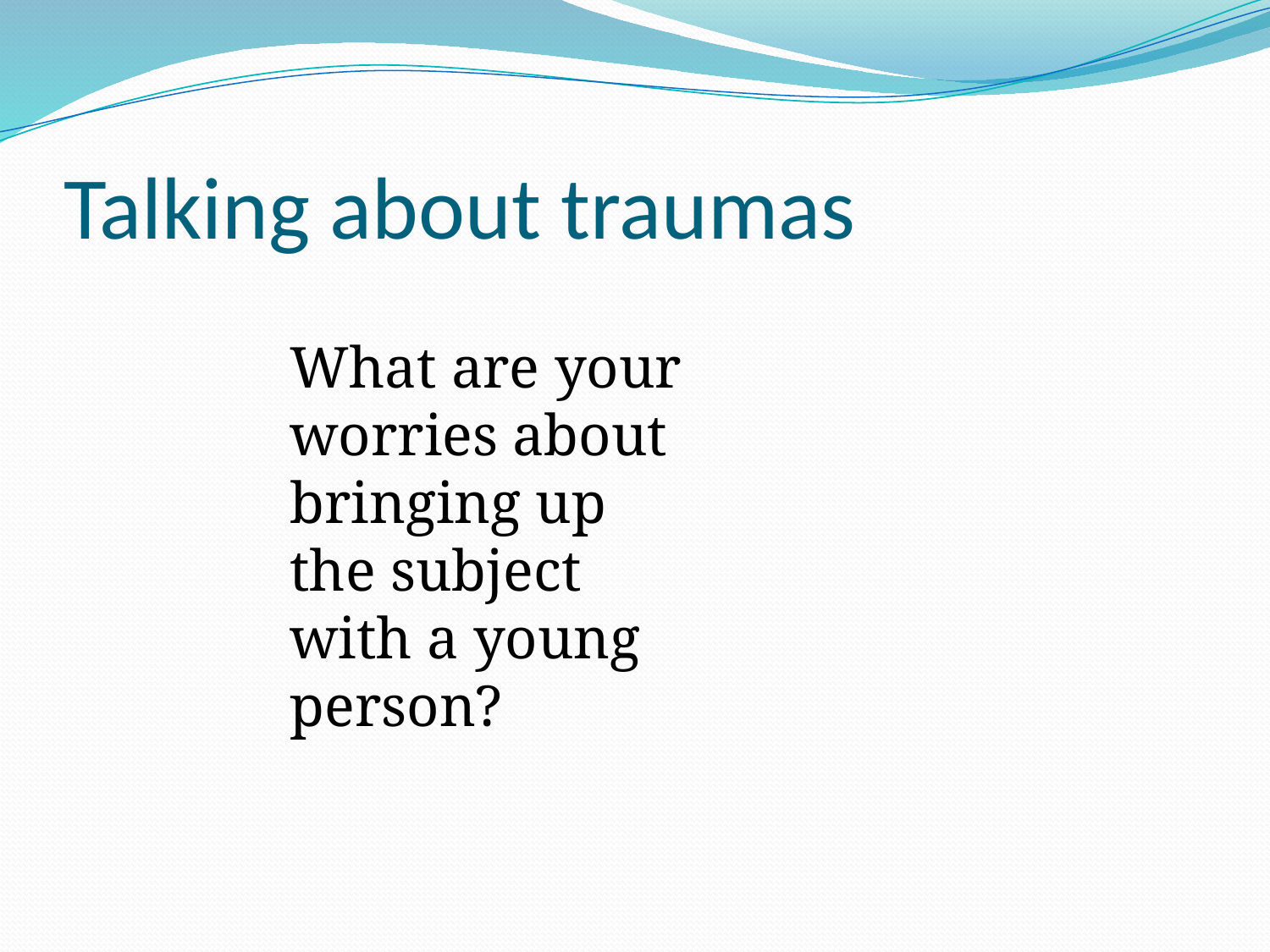

# Talking about traumas
What are your worries about bringing up the subject with a young person?

## Slide 15
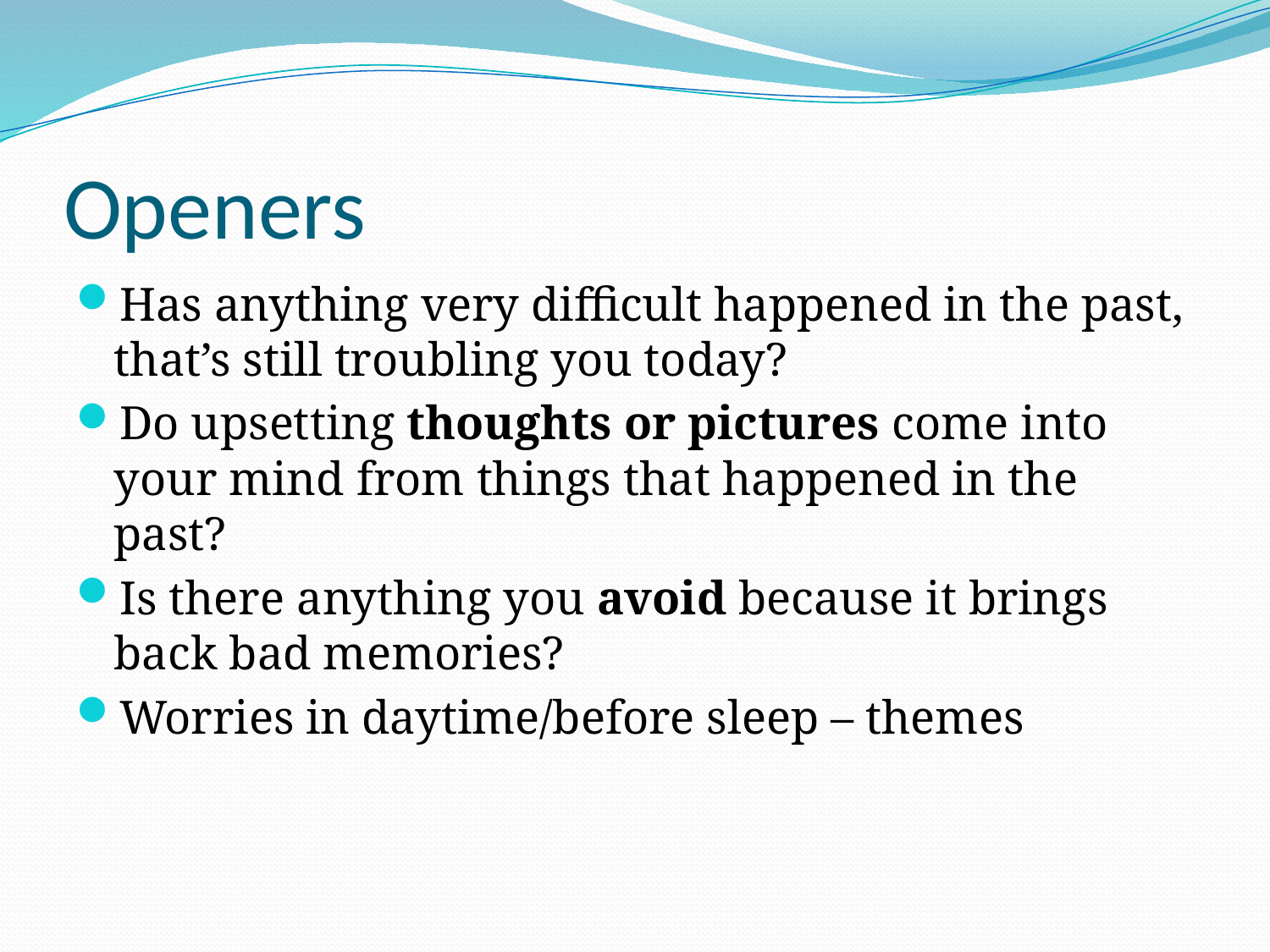

# Openers
Has anything very difficult happened in the past, that’s still troubling you today?
Do upsetting thoughts or pictures come into your mind from things that happened in the past?
Is there anything you avoid because it brings back bad memories?
Worries in daytime/before sleep – themes

## Slide 16
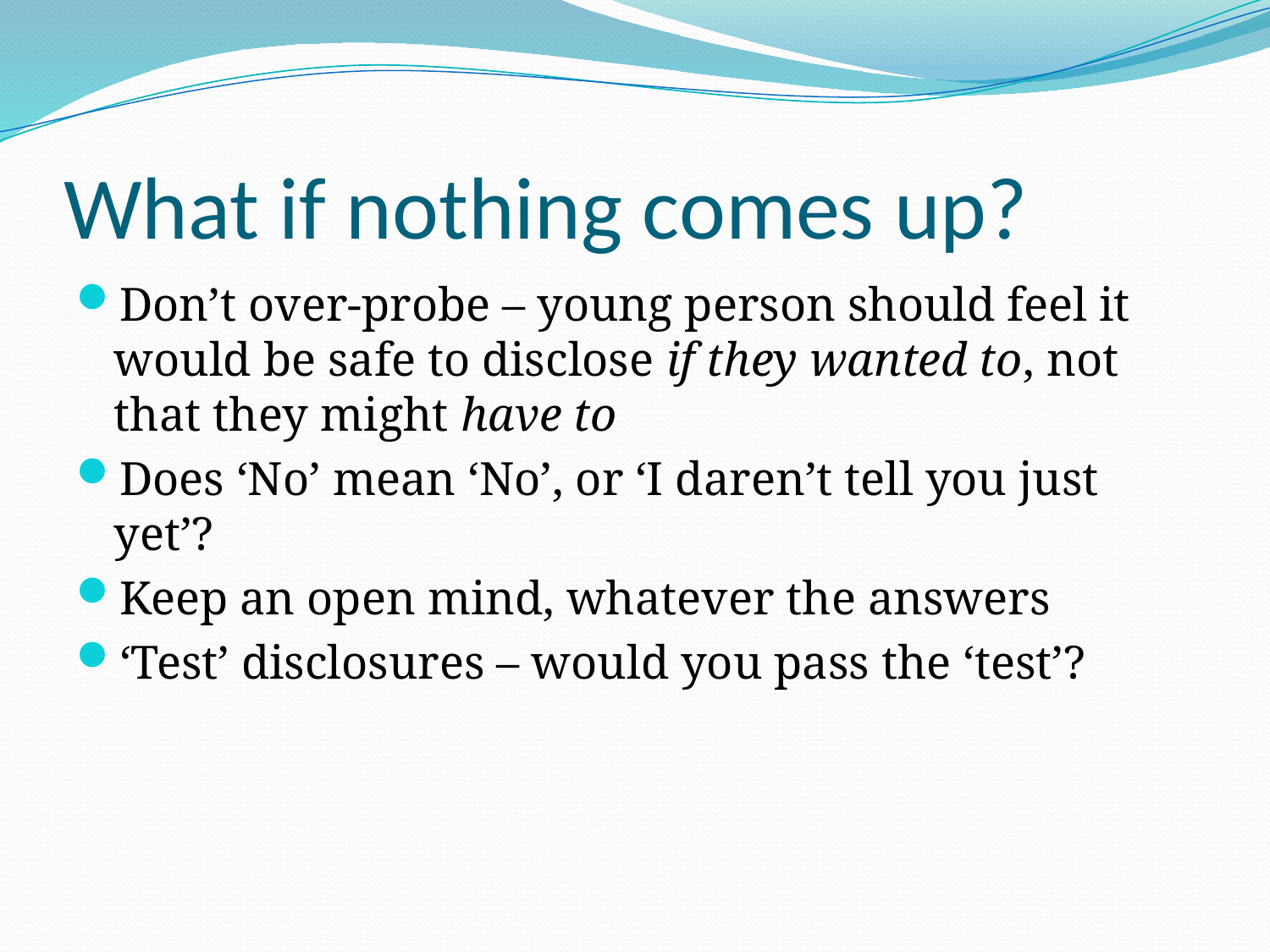

# What if nothing comes up?
Don’t over-probe – young person should feel it would be safe to disclose if they wanted to, not that they might have to
Does ‘No’ mean ‘No’, or ‘I daren’t tell you just yet’?
Keep an open mind, whatever the answers
‘Test’ disclosures – would you pass the ‘test’?

## Slide 17
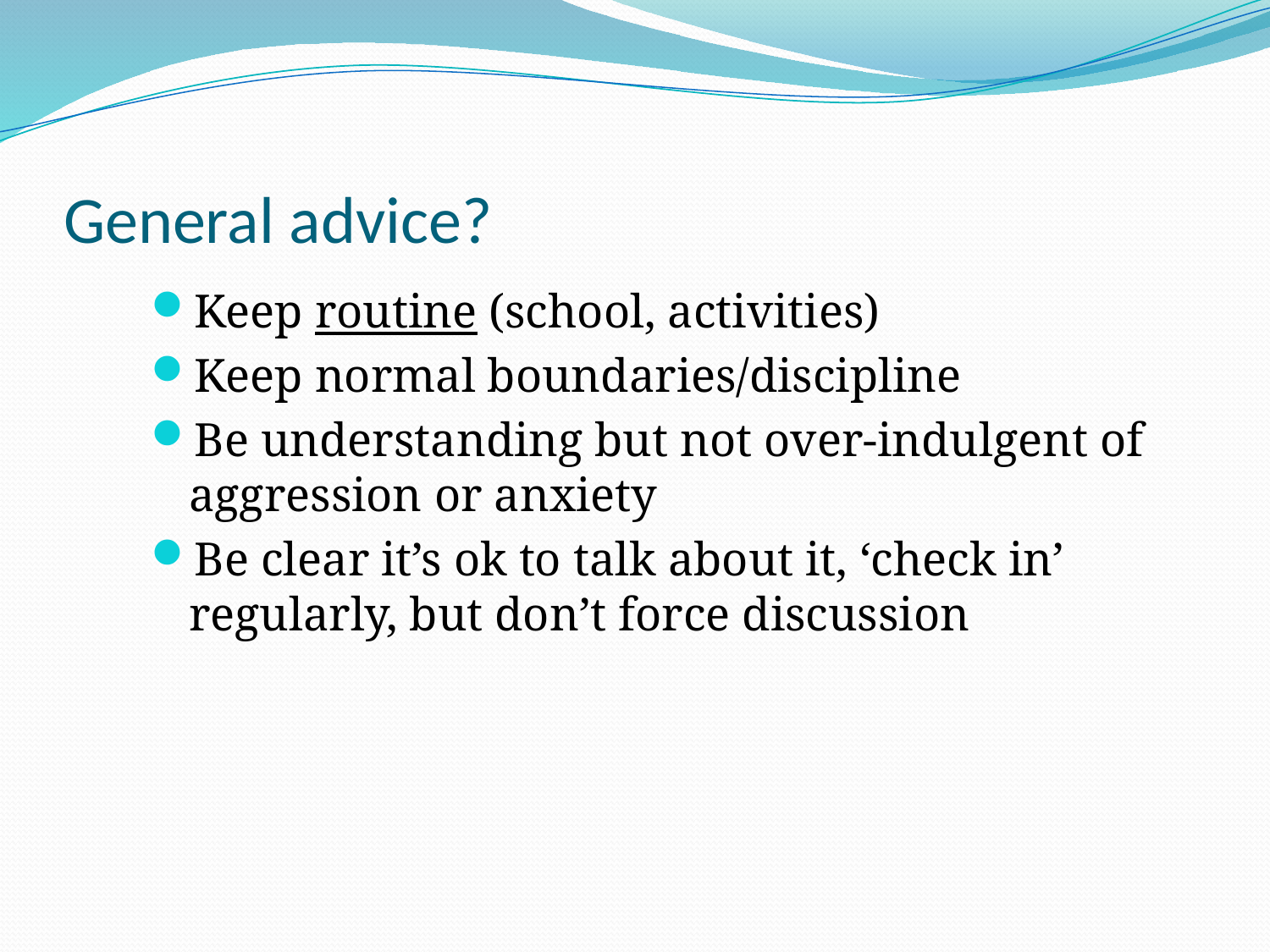

# General advice?
Keep routine (school, activities)
Keep normal boundaries/discipline
Be understanding but not over-indulgent of aggression or anxiety
Be clear it’s ok to talk about it, ‘check in’ regularly, but don’t force discussion

## Slide 18
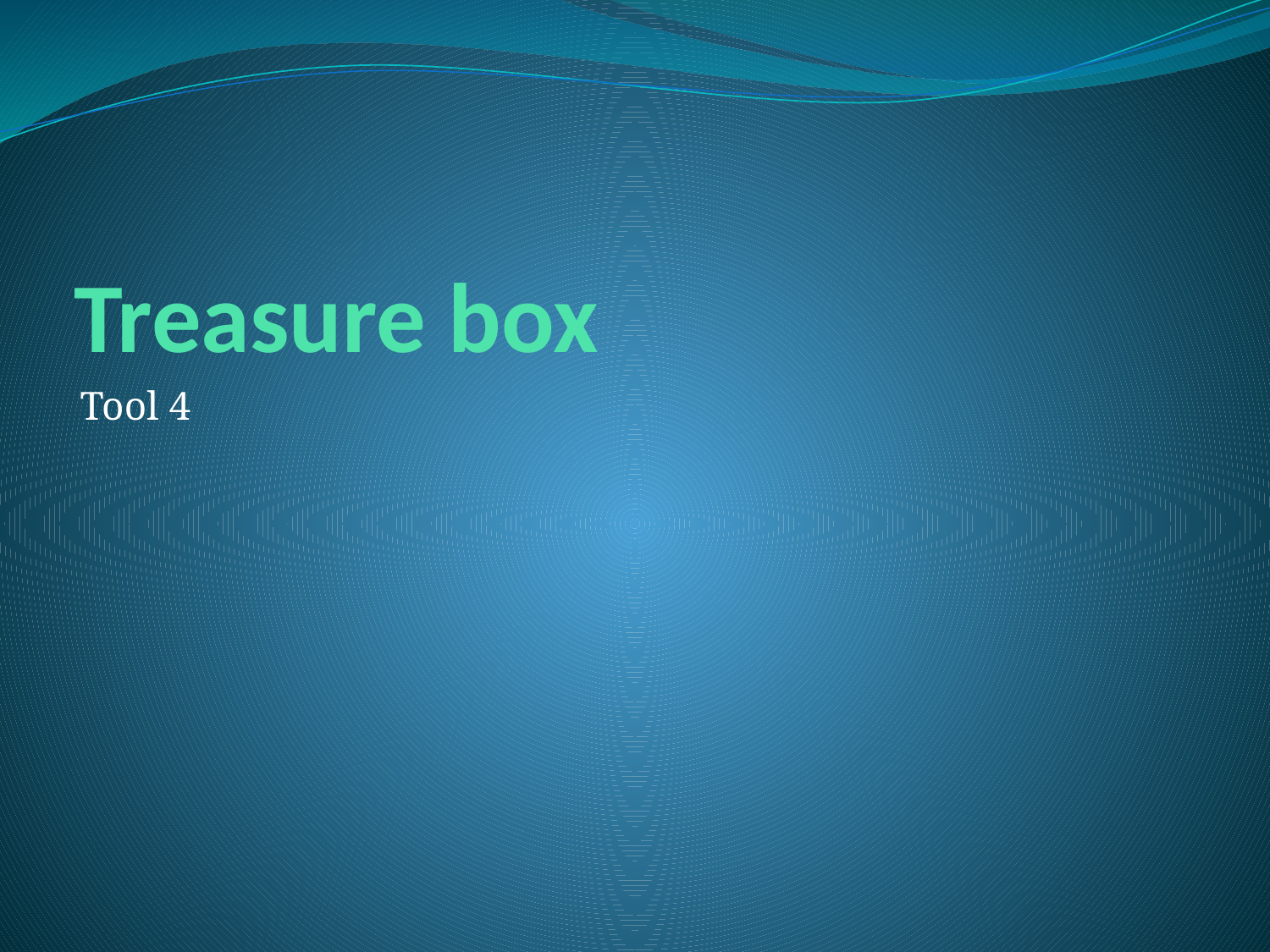

# Treasure box
Tool 4

## Slide 19
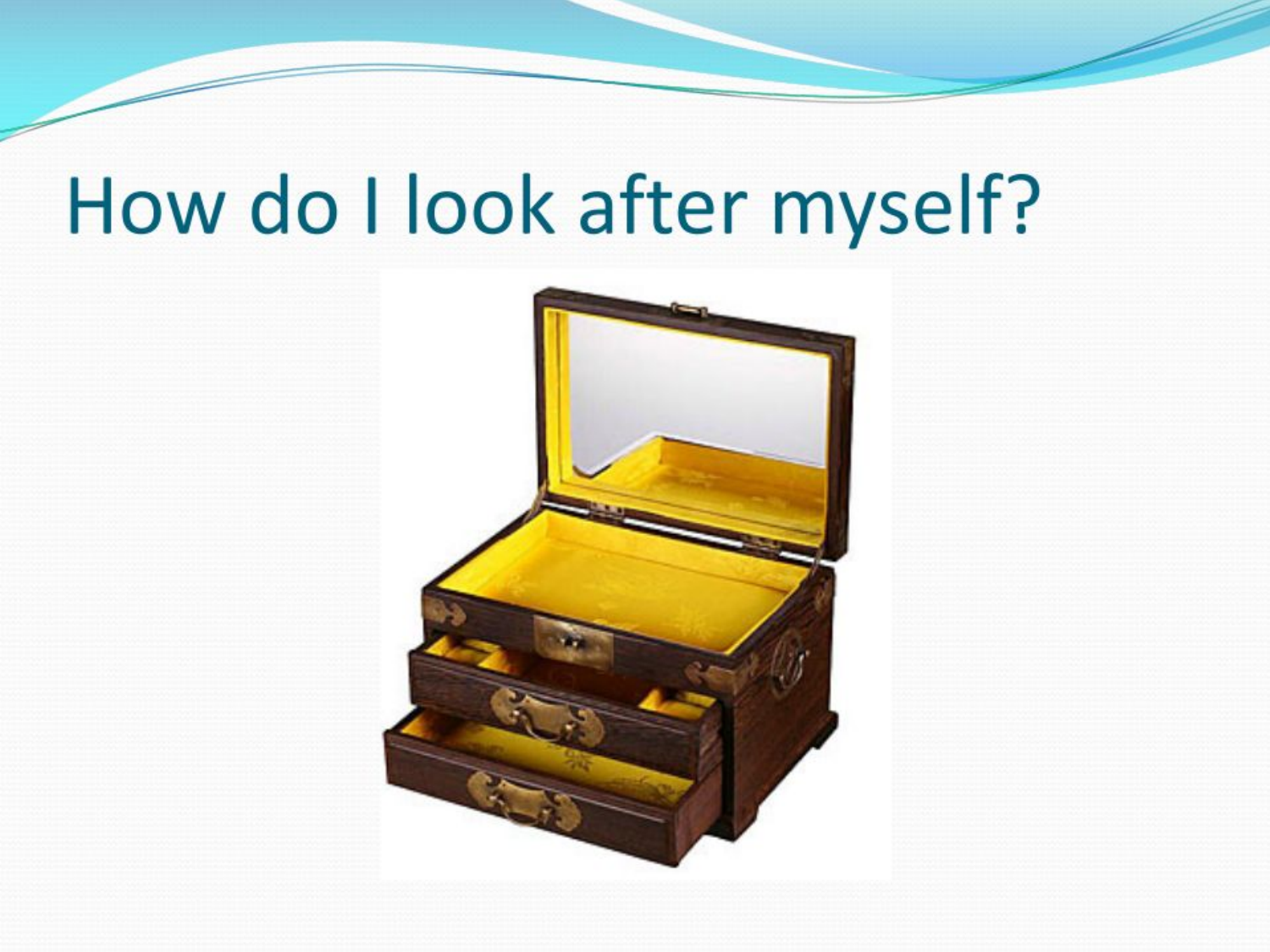

# How do I look after myself?

## Slide 20
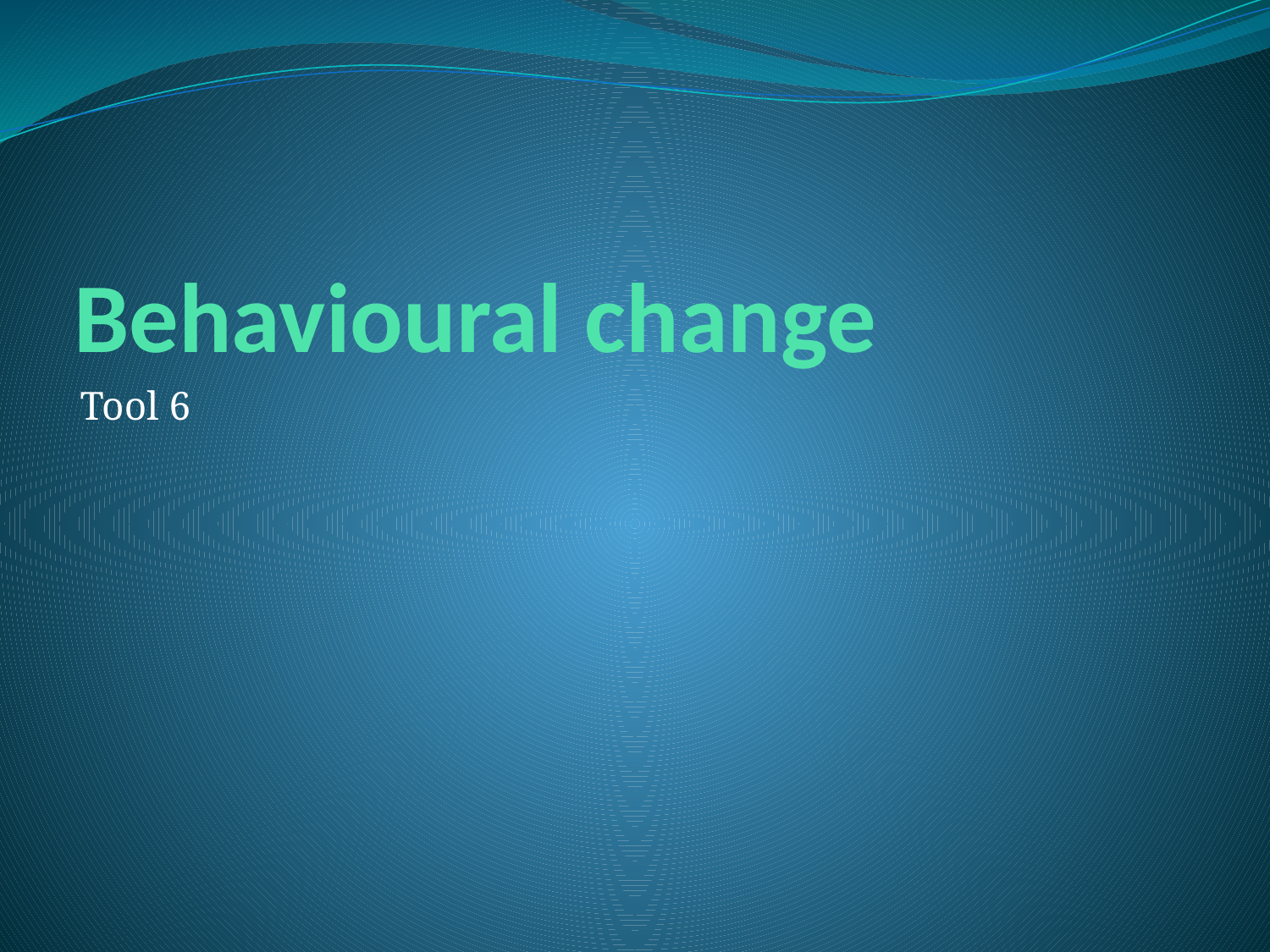

# Behavioural change
Tool 6

## Slide 21
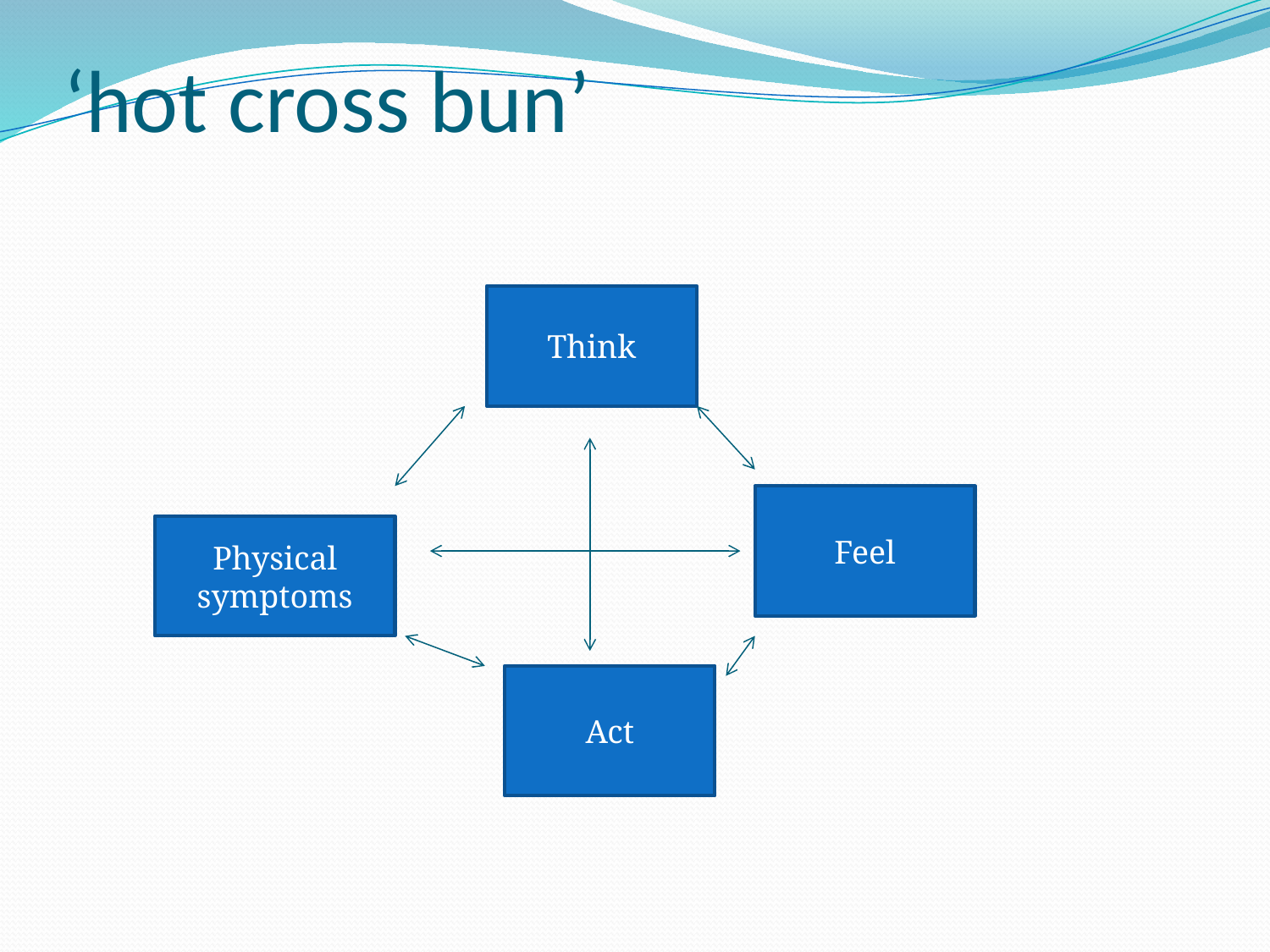

# ‘hot cross bun’
Think
Feel
Physical symptoms
Act

## Slide 22
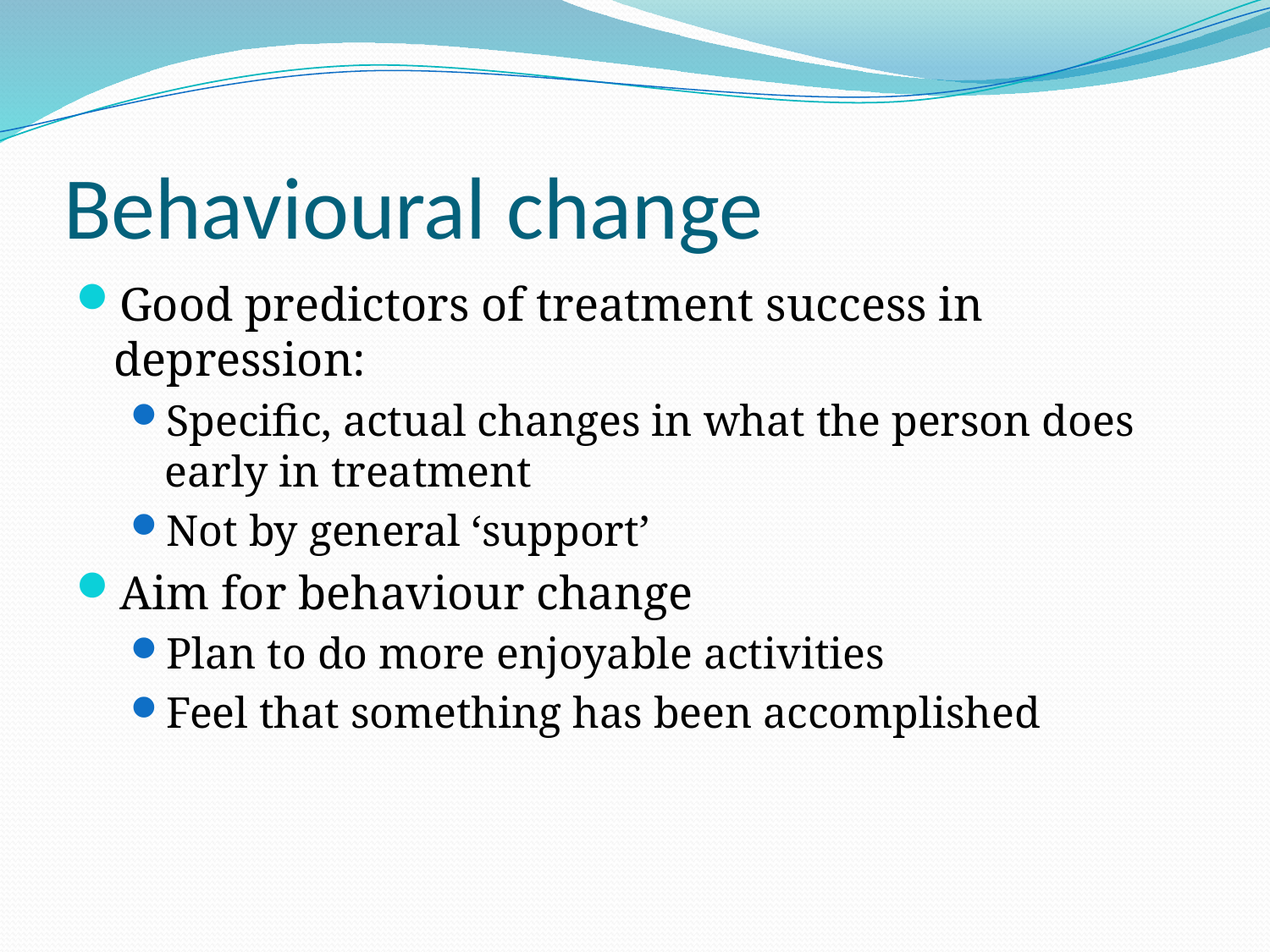

# Behavioural change
Good predictors of treatment success in depression:
Specific, actual changes in what the person does early in treatment
Not by general ‘support’
Aim for behaviour change
Plan to do more enjoyable activities
Feel that something has been accomplished

## Slide 23
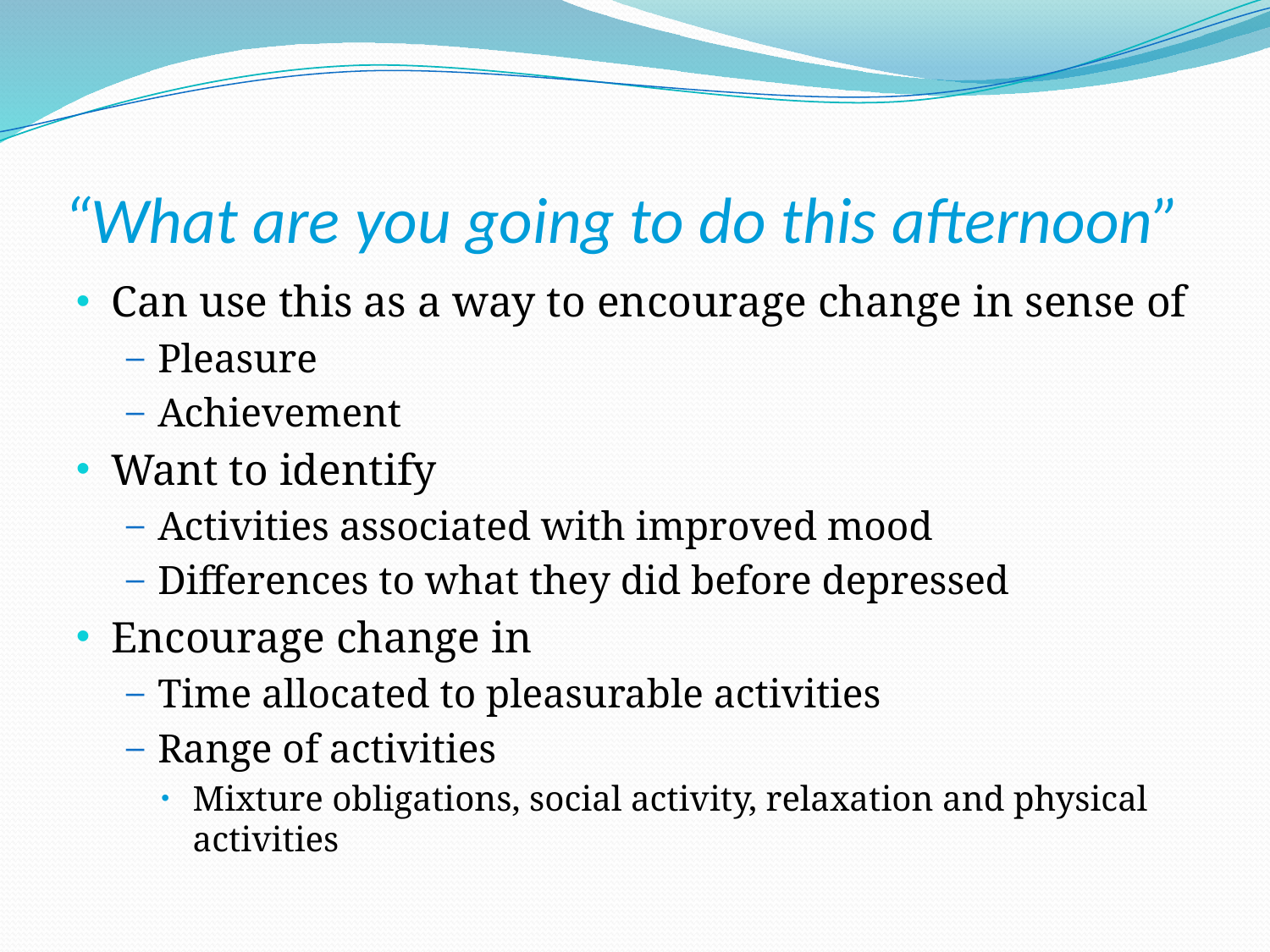

# “What are you going to do this afternoon”
Can use this as a way to encourage change in sense of
Pleasure
Achievement
Want to identify
Activities associated with improved mood
Differences to what they did before depressed
Encourage change in
Time allocated to pleasurable activities
Range of activities
Mixture obligations, social activity, relaxation and physical activities

## Slide 24
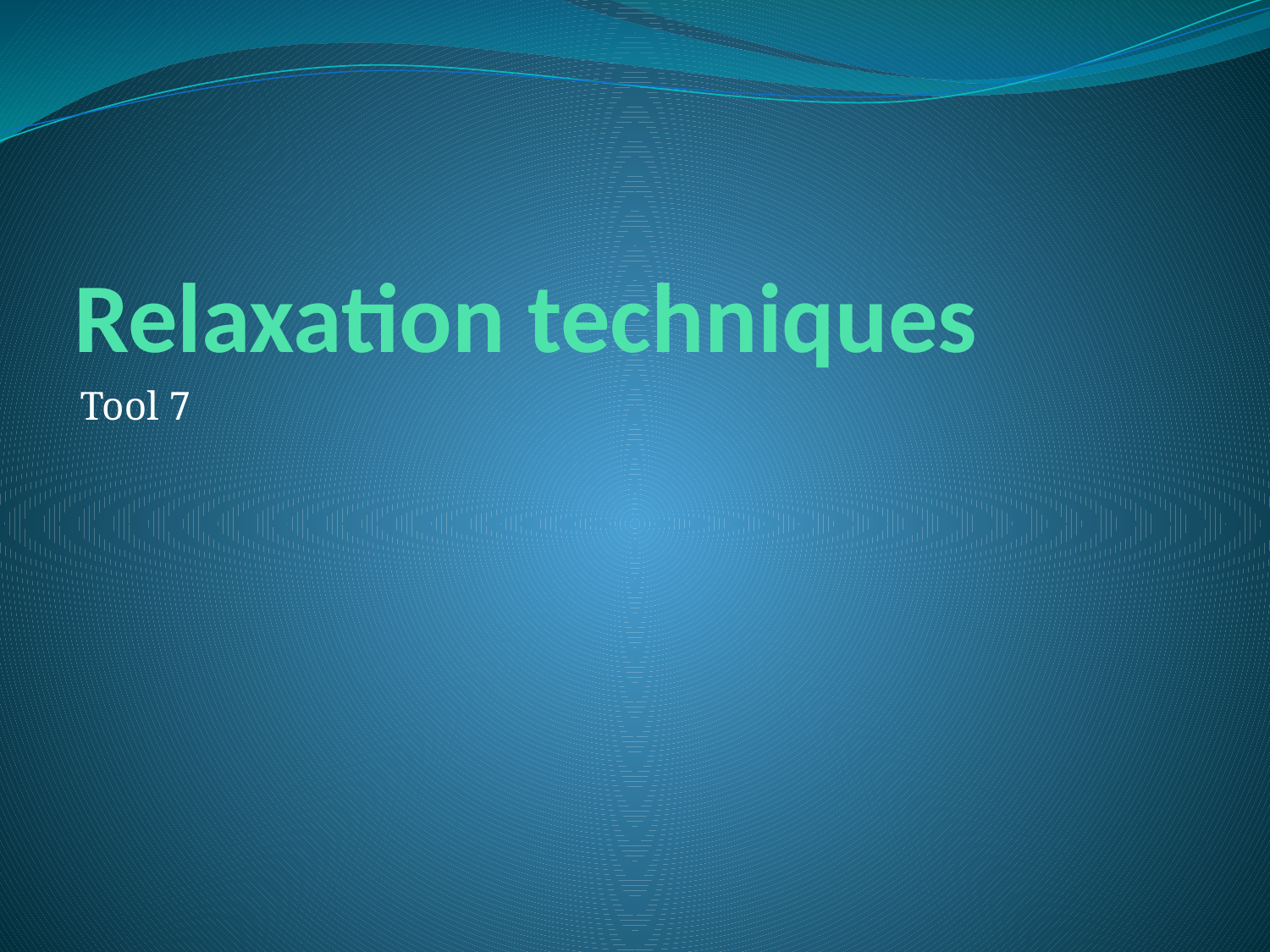

# Relaxation techniques
Tool 7

## Slide 25
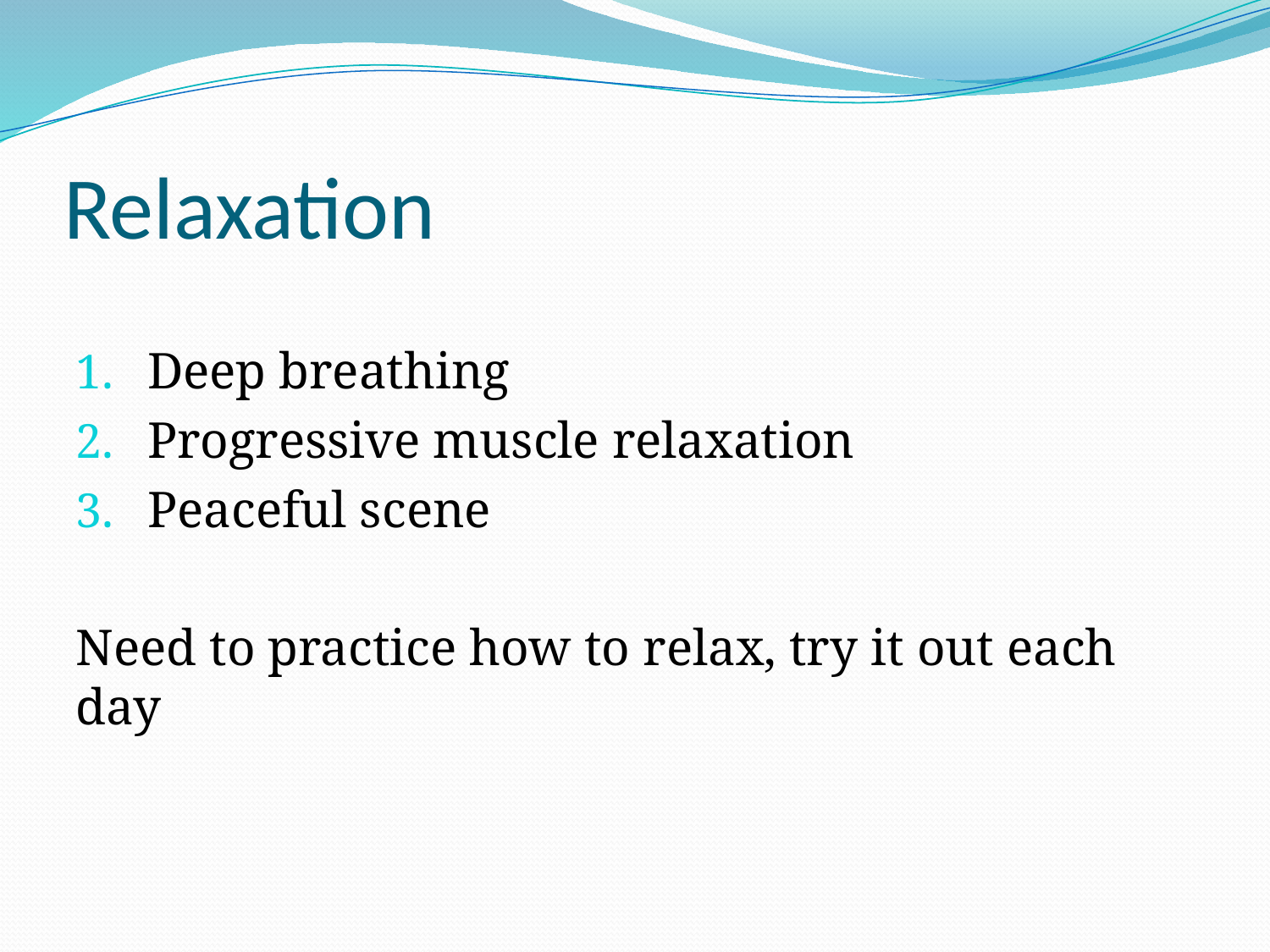

# Relaxation
Deep breathing
Progressive muscle relaxation
Peaceful scene
Need to practice how to relax, try it out each day

## Slide 26
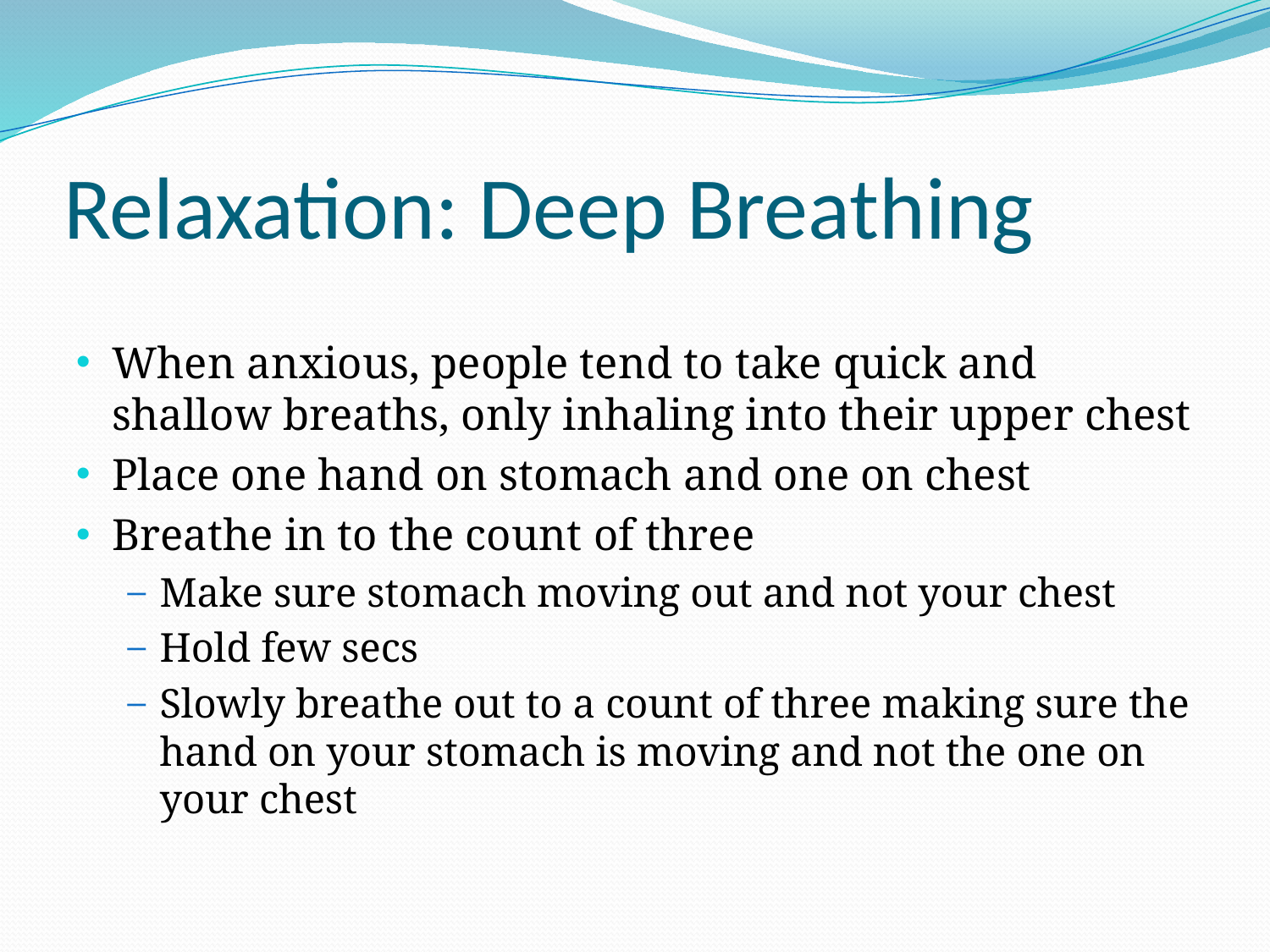

# Relaxation: Deep Breathing
When anxious, people tend to take quick and shallow breaths, only inhaling into their upper chest
Place one hand on stomach and one on chest
Breathe in to the count of three
Make sure stomach moving out and not your chest
Hold few secs
Slowly breathe out to a count of three making sure the hand on your stomach is moving and not the one on your chest

## Slide 27
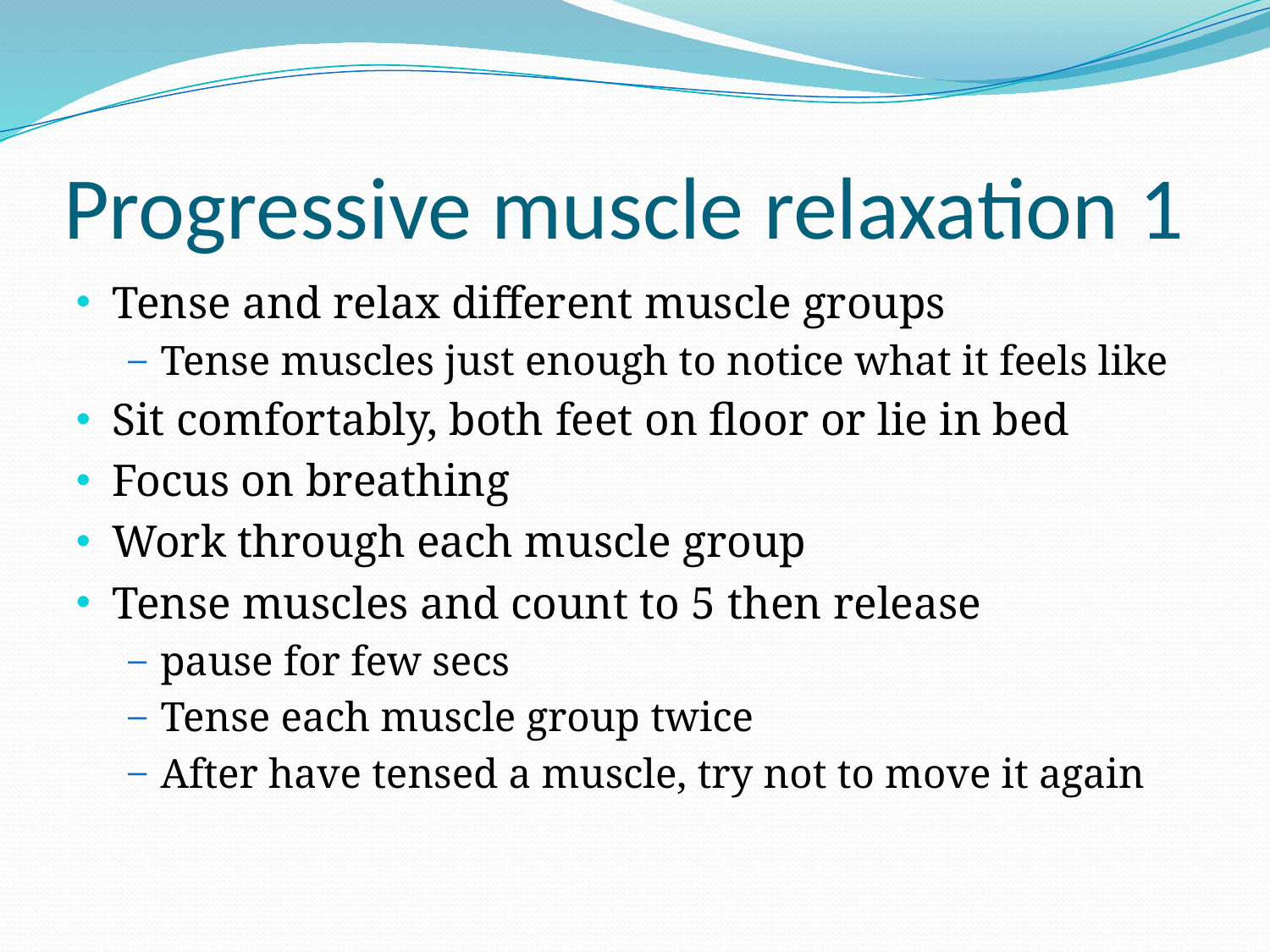

# Progressive muscle relaxation 1
Tense and relax different muscle groups
Tense muscles just enough to notice what it feels like
Sit comfortably, both feet on floor or lie in bed
Focus on breathing
Work through each muscle group
Tense muscles and count to 5 then release
pause for few secs
Tense each muscle group twice
After have tensed a muscle, try not to move it again

## Slide 28
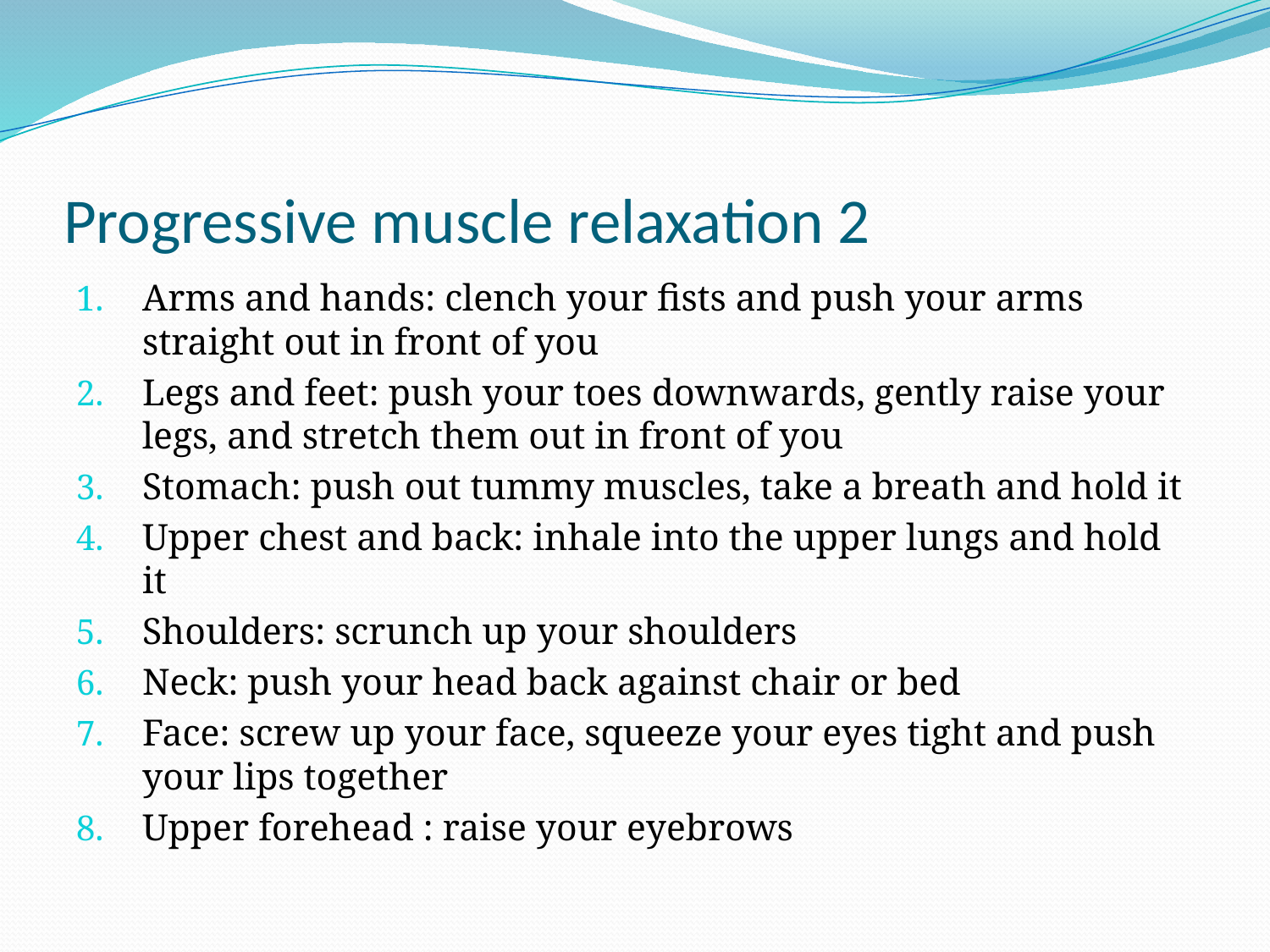

# Progressive muscle relaxation 2
Arms and hands: clench your fists and push your arms straight out in front of you
Legs and feet: push your toes downwards, gently raise your legs, and stretch them out in front of you
Stomach: push out tummy muscles, take a breath and hold it
Upper chest and back: inhale into the upper lungs and hold it
Shoulders: scrunch up your shoulders
Neck: push your head back against chair or bed
Face: screw up your face, squeeze your eyes tight and push your lips together
Upper forehead : raise your eyebrows

## Slide 29
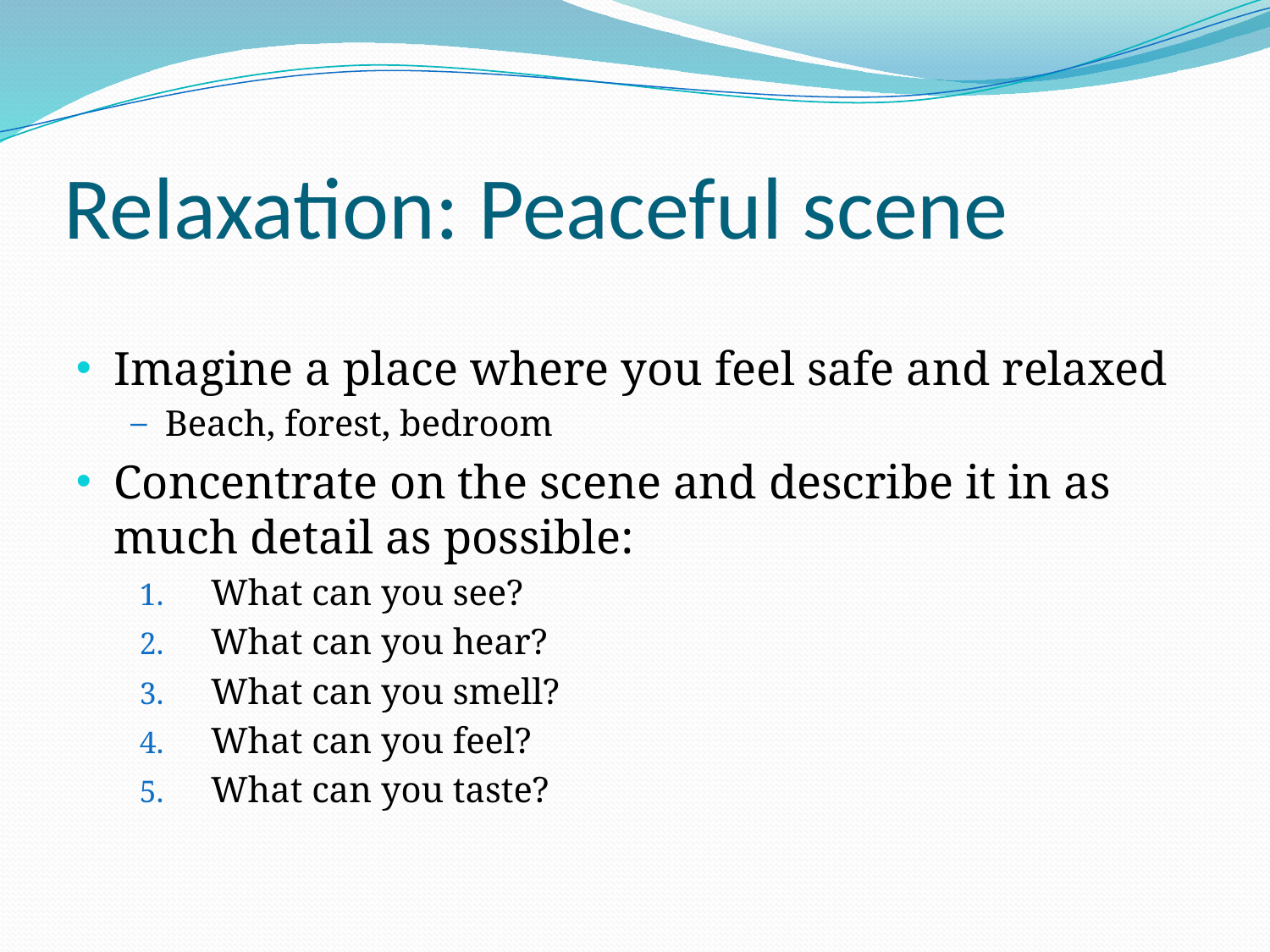

# Relaxation: Peaceful scene
Imagine a place where you feel safe and relaxed
Beach, forest, bedroom
Concentrate on the scene and describe it in as much detail as possible:
What can you see?
What can you hear?
What can you smell?
What can you feel?
What can you taste?

## Slide 30
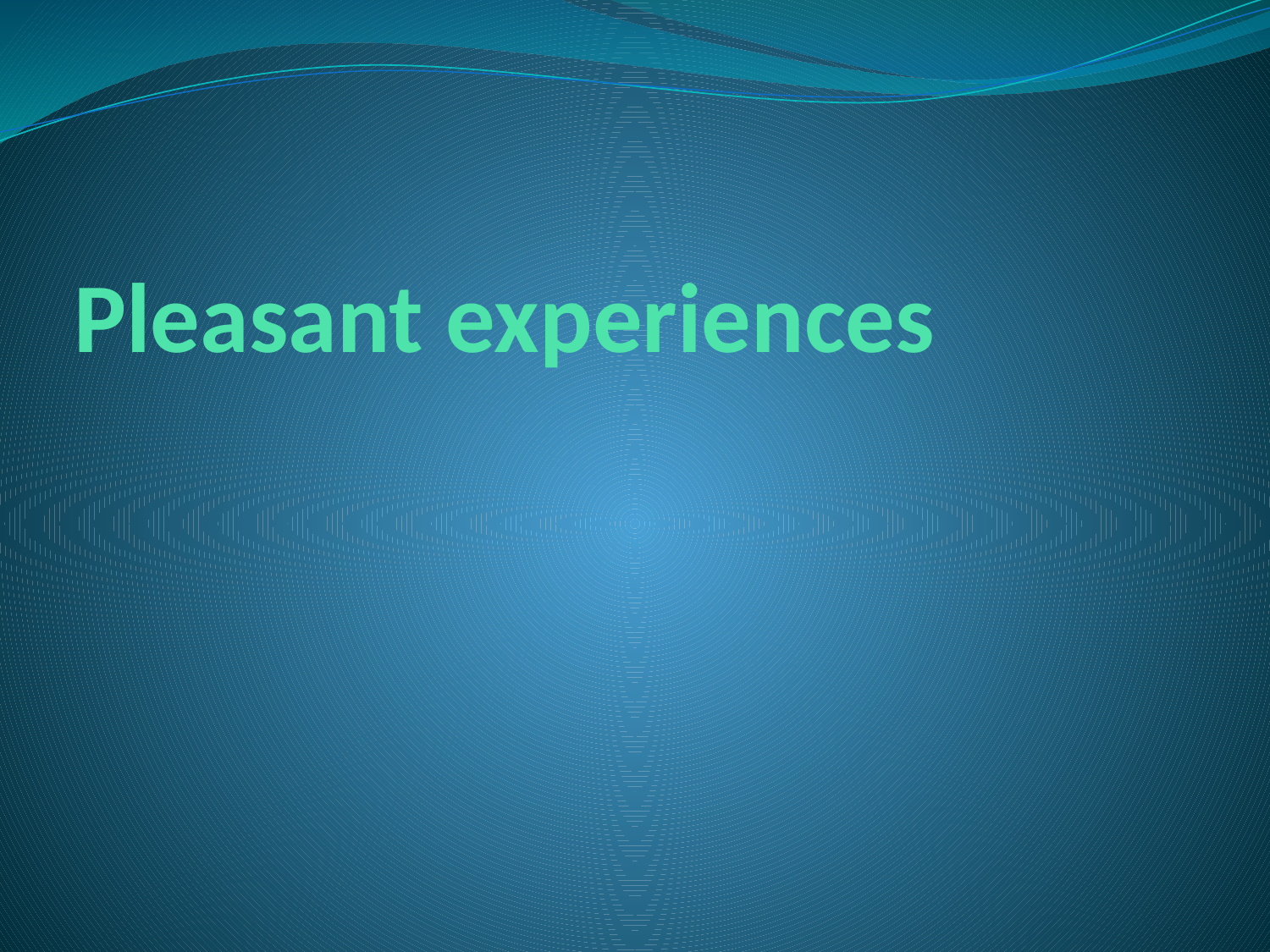

# Pleasant experiences

## Slide 31
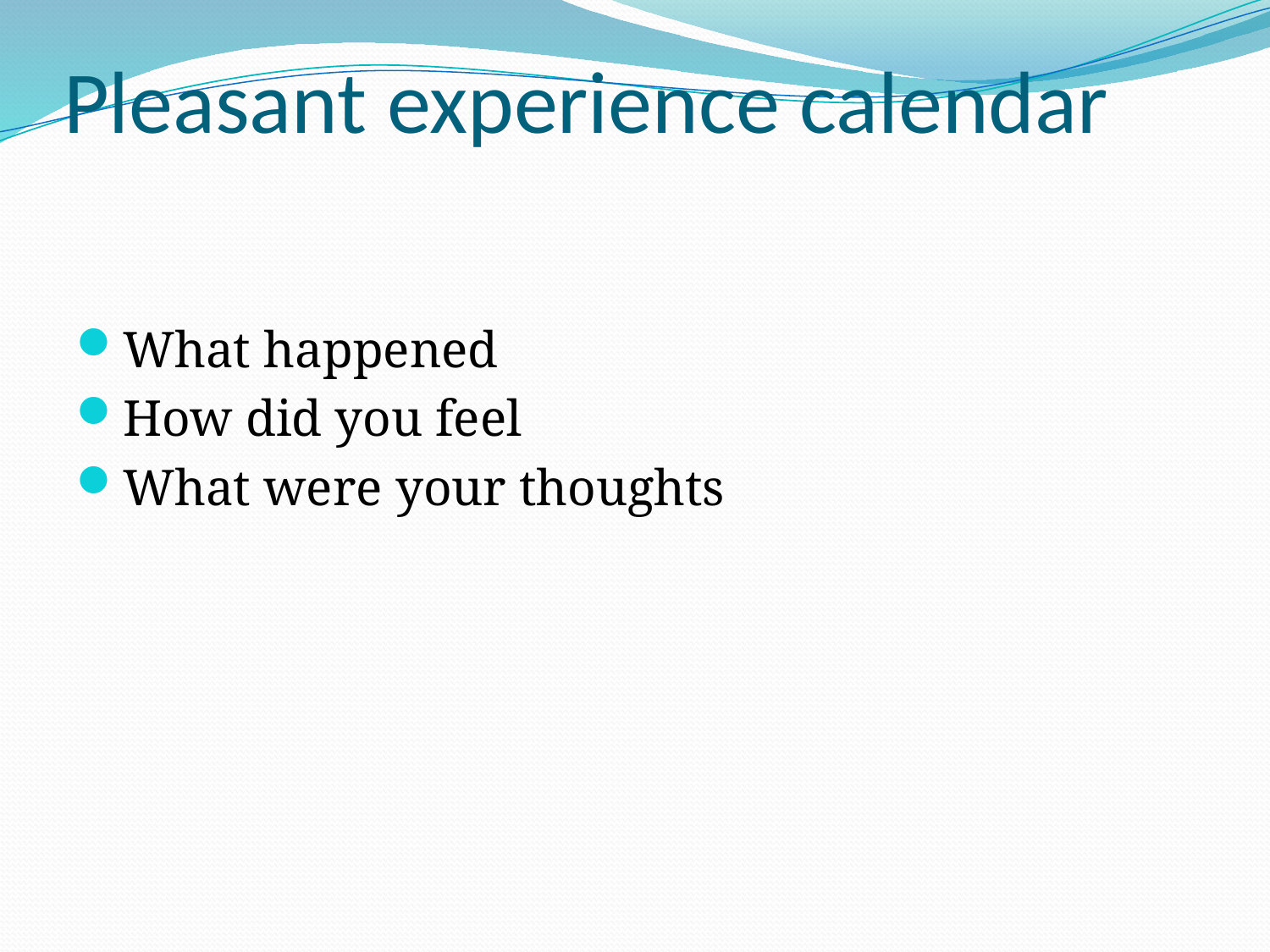

# Pleasant experience calendar
What happened
How did you feel
What were your thoughts

## Slide 32
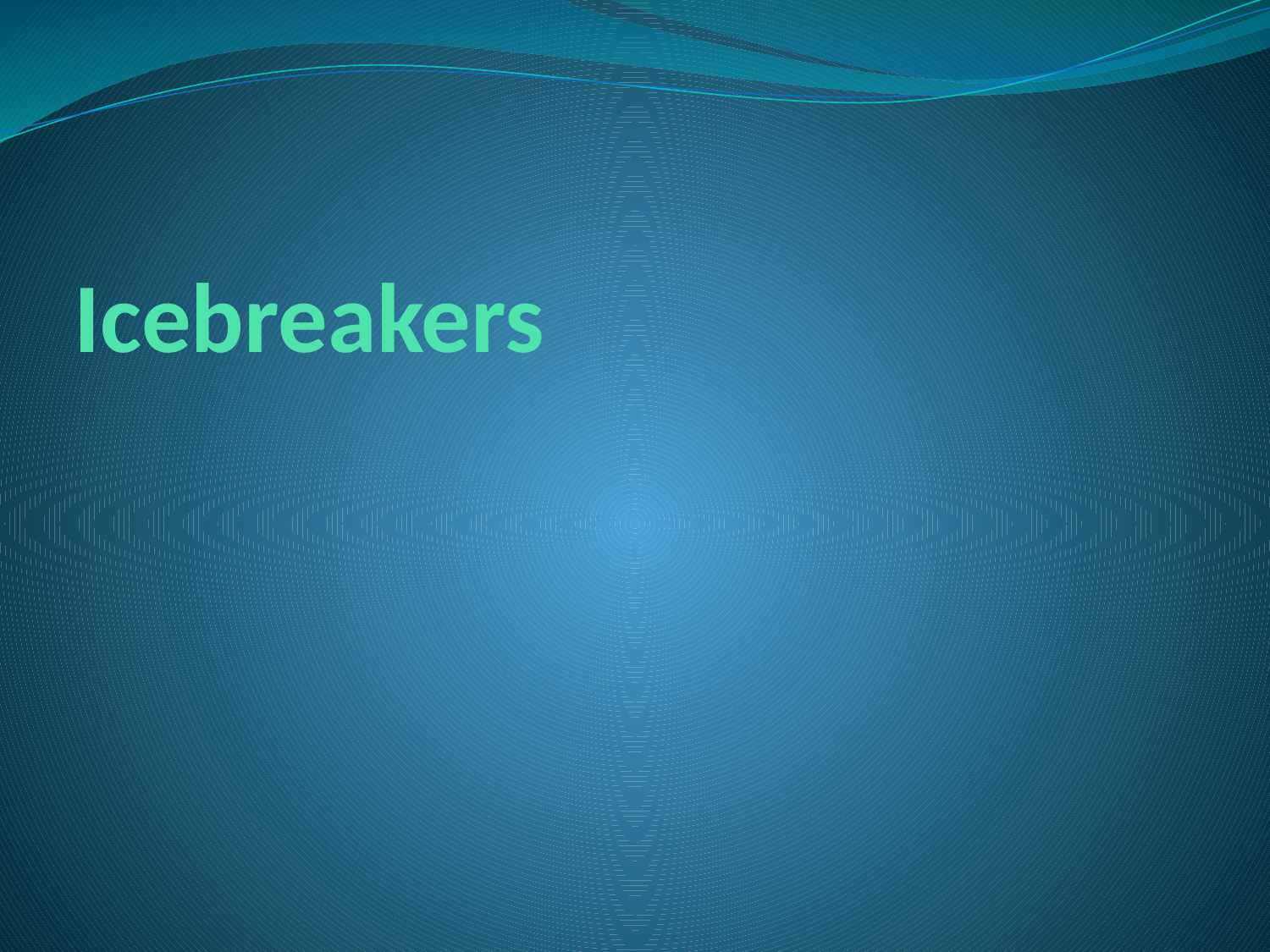

# Icebreakers

## Slide 33
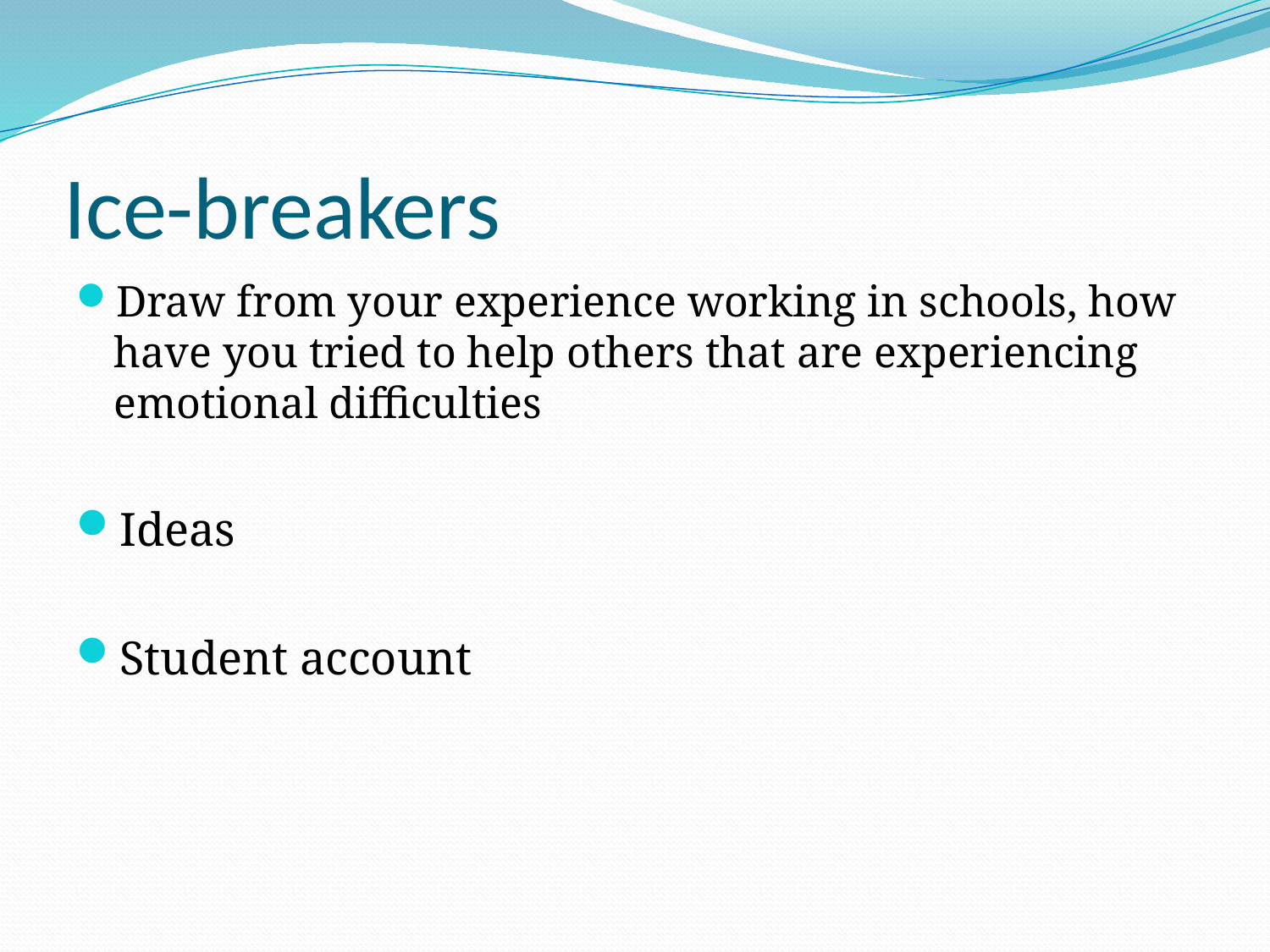

# Ice-breakers
Draw from your experience working in schools, how have you tried to help others that are experiencing emotional difficulties
Ideas
Student account

## Slide 34
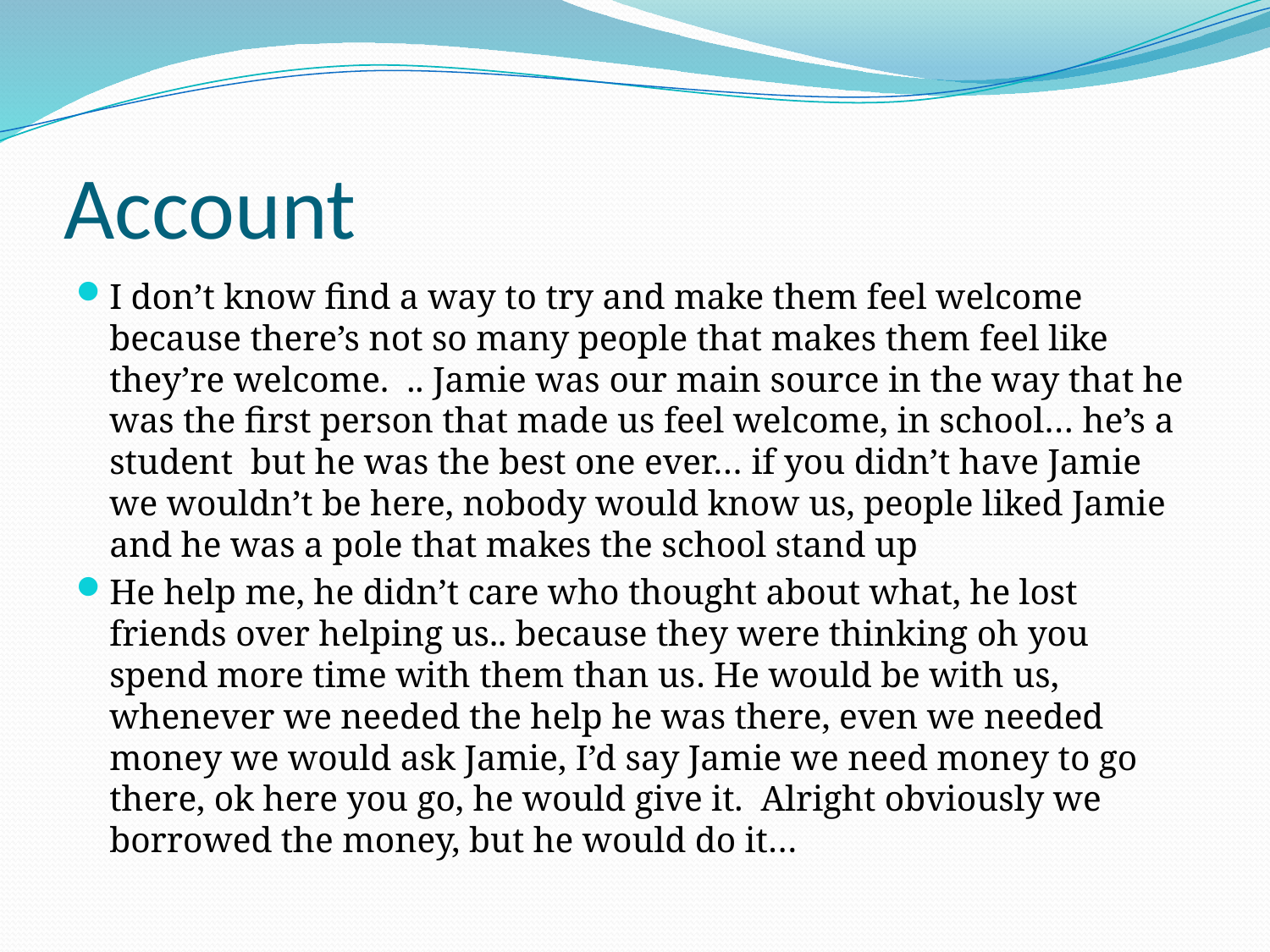

# Account
I don’t know find a way to try and make them feel welcome because there’s not so many people that makes them feel like they’re welcome. .. Jamie was our main source in the way that he was the first person that made us feel welcome, in school… he’s a student but he was the best one ever… if you didn’t have Jamie we wouldn’t be here, nobody would know us, people liked Jamie and he was a pole that makes the school stand up
He help me, he didn’t care who thought about what, he lost friends over helping us.. because they were thinking oh you spend more time with them than us. He would be with us, whenever we needed the help he was there, even we needed money we would ask Jamie, I’d say Jamie we need money to go there, ok here you go, he would give it. Alright obviously we borrowed the money, but he would do it…

## Slide 35
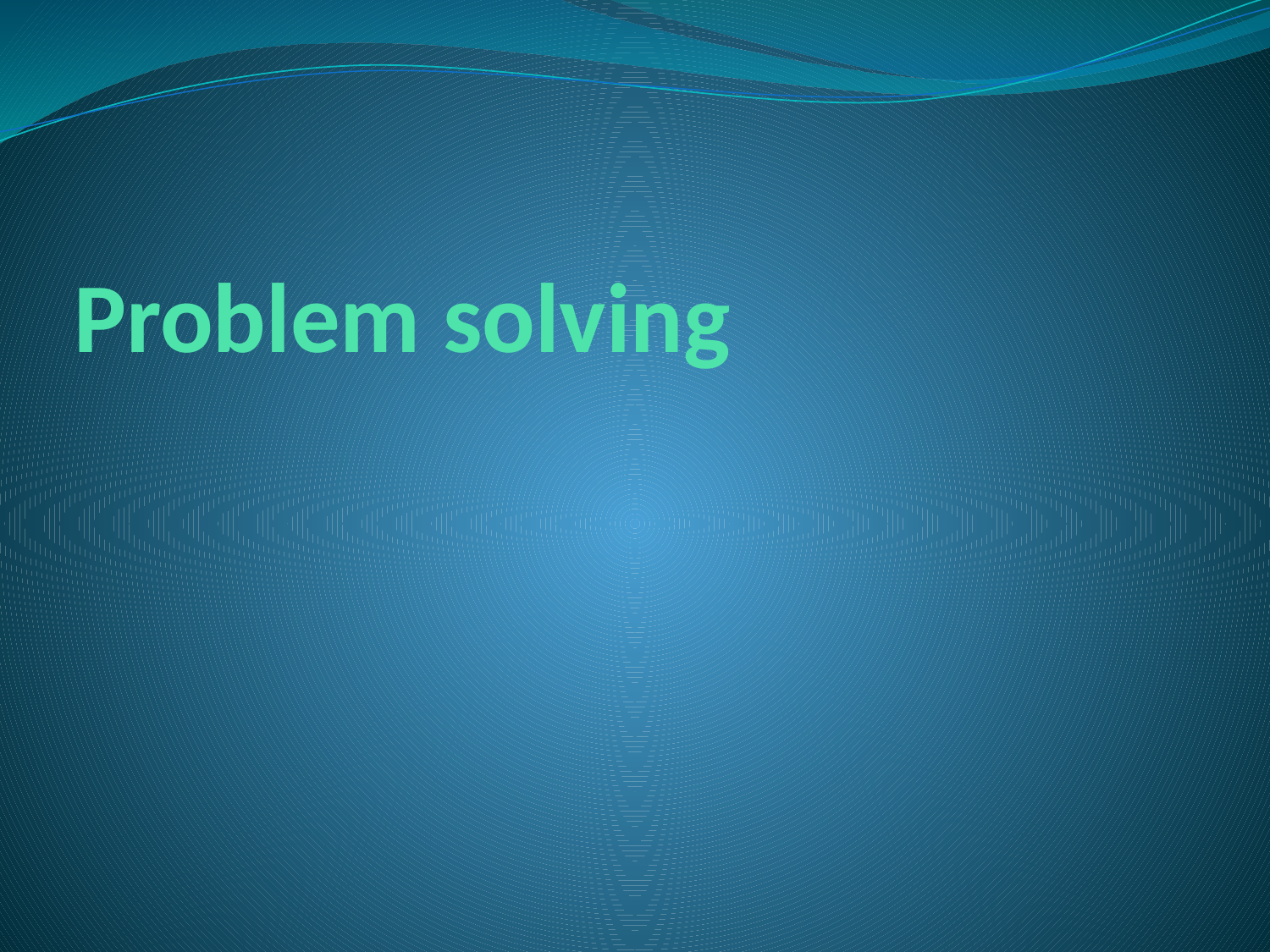

# Problem solving

## Slide 36
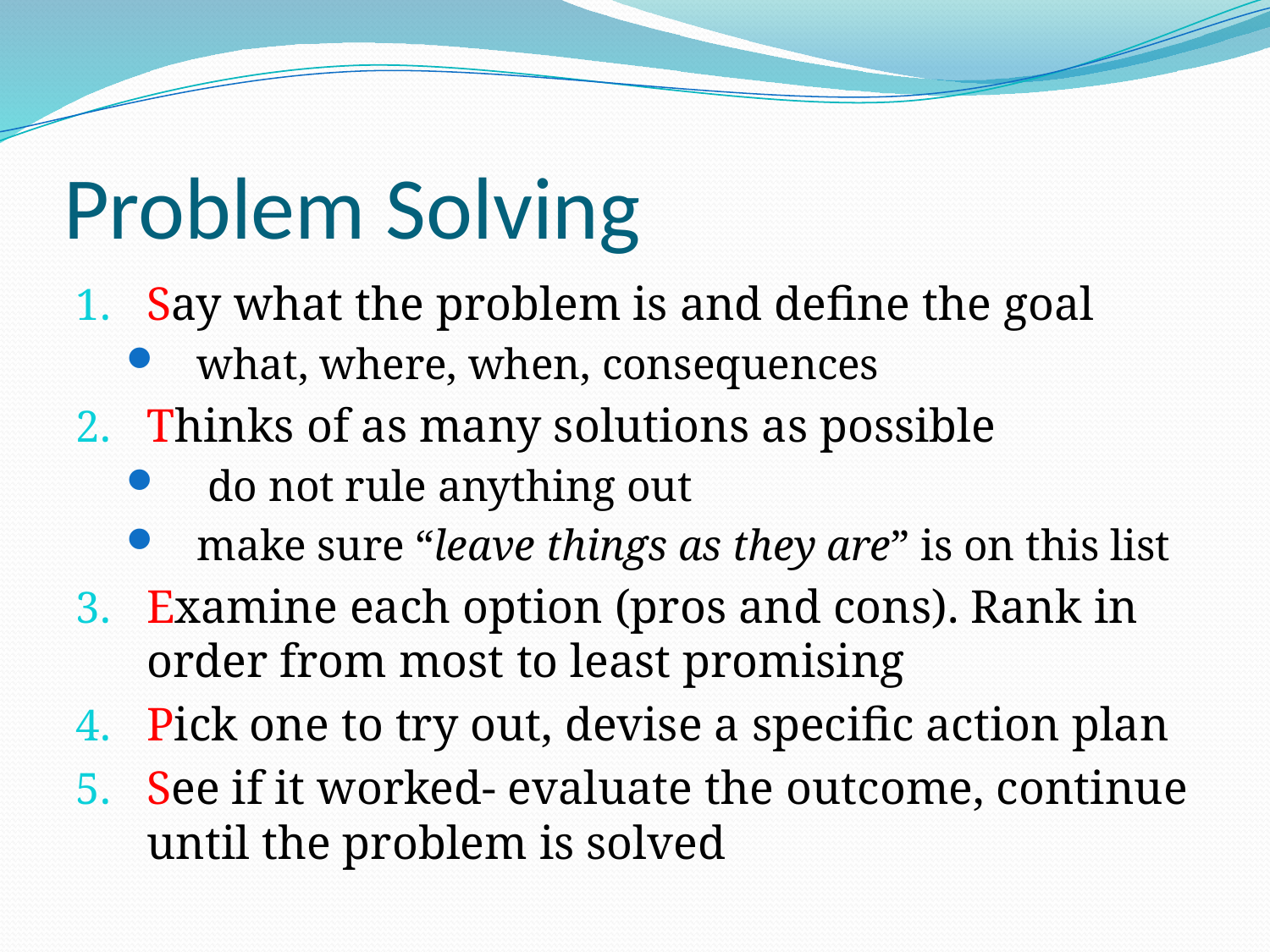

# Problem Solving
Say what the problem is and define the goal
what, where, when, consequences
Thinks of as many solutions as possible
 do not rule anything out
make sure “leave things as they are” is on this list
Examine each option (pros and cons). Rank in order from most to least promising
Pick one to try out, devise a specific action plan
See if it worked- evaluate the outcome, continue until the problem is solved

## Slide 37
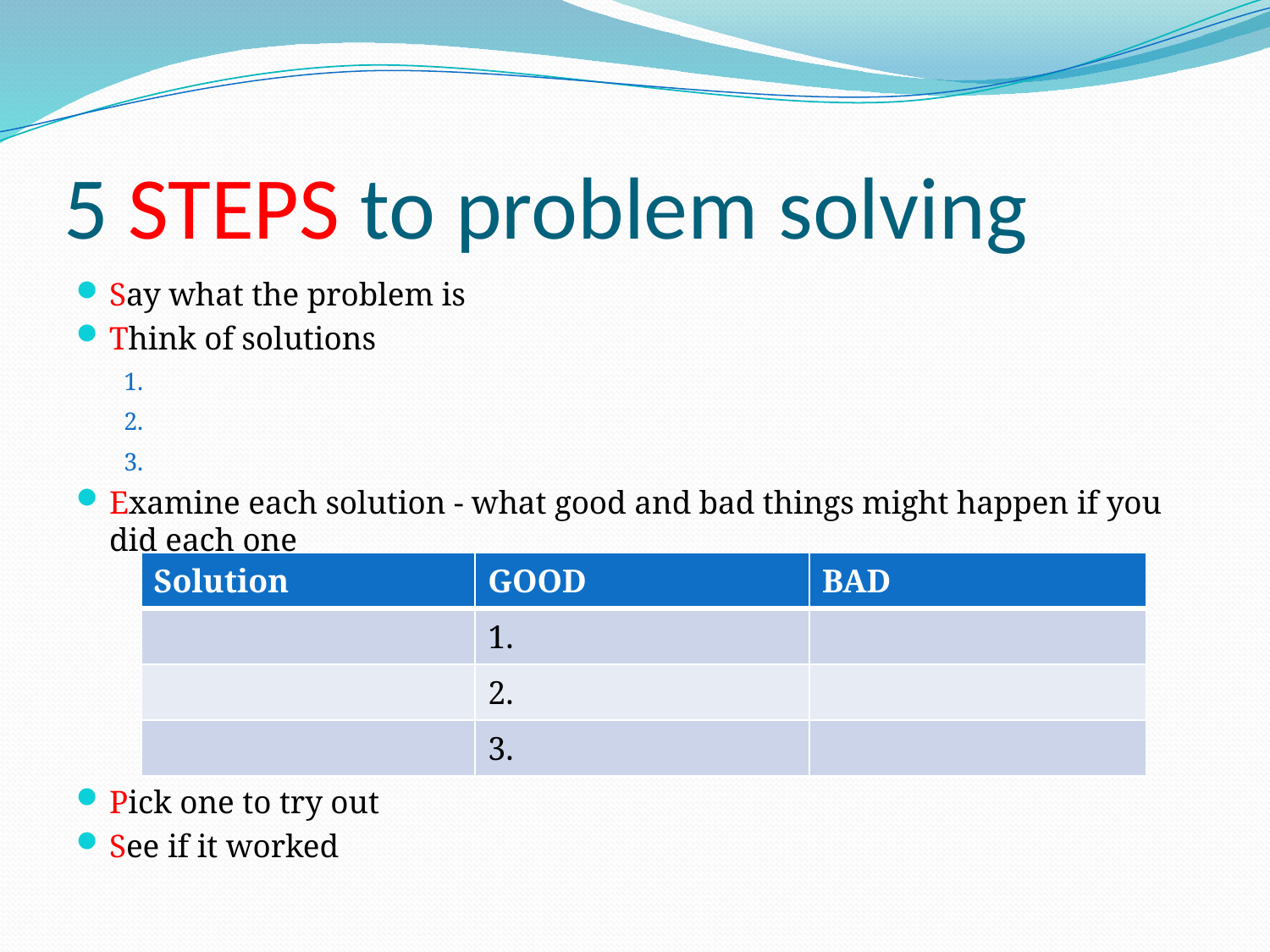

# 5 STEPS to problem solving
Say what the problem is
Think of solutions
Examine each solution - what good and bad things might happen if you did each one
Pick one to try out
See if it worked
| Solution | GOOD | BAD |
| --- | --- | --- |
| | 1. | |
| | 2. | |
| | 3. | |
